# Triple base editor catalyzes saturation mutation of adenine, cytidine, and guanine

**DOI:** 10.1093/nar/gkaf1423

**Published:** 2026-01-14

**Authors:** Youming Wu, Jinxin Wang, Ziyi Zhang, Mengyu Shang, Shuping Wang, Yinuo Li, Guangyu Li, Shuangshuang Lu, Kaiyuan Ji, Xiaoyue Wang, Xiaohui Zhang

**Affiliations:** State Key Laboratory of Common Mechanism Research for Major Diseases, Suzhou Institute of Systems Medicine, Chinese Academy of Medical Sciences and Peking Union Medical College, Suzhou 215123, China; State Key Laboratory of Common Mechanism Research for Major Diseases, Suzhou Institute of Systems Medicine, Chinese Academy of Medical Sciences and Peking Union Medical College, Suzhou 215123, China; Department of Biochemistry and Molecular Biology, School of Basic Medical Sciences, Zhengzhou University, Zhengzhou 450052, China; State Key Laboratory of Common Mechanism Research for Major Diseases, Suzhou Institute of Systems Medicine, Chinese Academy of Medical Sciences and Peking Union Medical College, Suzhou 215123, China; State Key Laboratory of Common Mechanism Research for Major Diseases, Suzhou Institute of Systems Medicine, Chinese Academy of Medical Sciences and Peking Union Medical College, Suzhou 215123, China; State Key Laboratory of Common Mechanism Research for Major Diseases, Suzhou Institute of Systems Medicine, Chinese Academy of Medical Sciences and Peking Union Medical College, Suzhou 215123, China; State Key Laboratory of Common Mechanism Research for Major Diseases, Center for Bioinformatics, National Infrastructures for Translational Medicine, Institute of Clinical Medicine and Peking Union Medical College Hospital, Chinese Academy of Medical Sciences and Peking Union Medical College, Beijing 100730, China; Department of Biochemistry and Molecular Biology, School of Basic Medical Sciences, Zhengzhou University, Zhengzhou 450052, China; Guangzhou Key Laboratory of Maternal-Fetal Medicine, Institute of Reproductive Health and Perinatology, Guangzhou Women and Children’s Medical Center, Guangzhou Medical University, Guangzhou 511400, China; State Key Laboratory of Common Mechanism Research for Major Diseases, Center for Bioinformatics, National Infrastructures for Translational Medicine, Institute of Clinical Medicine and Peking Union Medical College Hospital, Chinese Academy of Medical Sciences and Peking Union Medical College, Beijing 100730, China; State Key Laboratory of Common Mechanism Research for Major Diseases, Suzhou Institute of Systems Medicine, Chinese Academy of Medical Sciences and Peking Union Medical College, Suzhou 215123, China

## Abstract

Current base editors act on a maximum of two base substrates and generate limited base conversions or transversions, hindering their applicability for inducing DNA sequence diversity. Here, we engineered a triple base editor (named ACG-BEs) using a fusion of adenine base editor with high A/C catalytic activity and evolved N-methylpurine DNA glycosylase. ACG-BEs enables efficient, multiplexed saturation mutagenesis across adenine (A), cytosine (C), and guanine (G), achieving conversion efficiencies of up to 80.5% for A-to-G/C/T, 75.8% for C-to-T/G/A, and 63.4% for G-to-C/T/A in HEK293T cells. Leveraging ACG-BEs, we identify novel mutations in the *HBG1/2* promoter region that confer efficient activation of γ-globin expression in HUDEP-2 cells—a promising advancement for therapeutic strategies targeting hemoglobinopathies. These findings highlight ACG-BEs as a cutting-edge platform for multiplexed saturation mutagenesis, offering broad applications in genetic screening and therapeutic base mutation introduction through enhanced DNA sequence diversity.

## Graphical Abstract

**Graphical Abstract:**
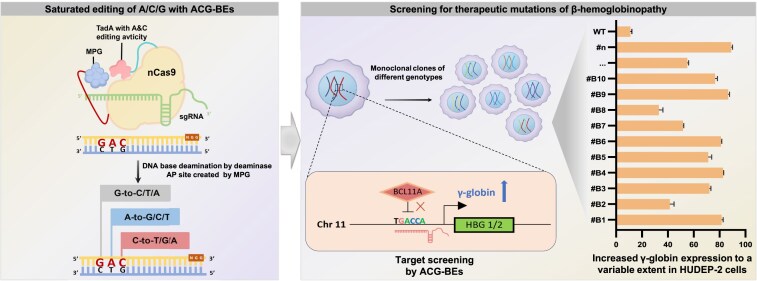

## Introduction

DNA base editors, which enable the precise and efficient catalysis of base conversions or transversions in genomic DNA, are powerful genome-editing tools for gene therapy, disease animal models, crop genetics and breeding, etc. [1]. The base editors, including single base editors and dual base editors, can also produce DNA sequence diversity by randomly inducing base conversions or transversions within editing windows (typically 3–8 nt) while performing base editing. This feature allows them to have great potential applications, such as genetic screening, directed evolution, and lineage tracing, etc. [2].

Thus far, base editors have accelerated research in genetic screening by inducing DNA sequence diversity in a given region of the genome and made many important research advances [3]. For example, two early studies by Ma *et al.* and Hess *et al.* reported that combining CRISPR/Cas9 with AID through fusion or recruitment allows the rapid generation of DNA-diverse variants for gain-of-function screens [4, 5]. Later, multiple studies have reported that combining ABE or CBE and single-guide RNAs (sgRNA) libraries established high-throughput screening platforms for specific purposes, such as functional point mutation screening for DNA damage response [6], studying interferon-γ signaling in cancer [7], enhancing the antitumor effect of human primary T cells [8], Genome-wide interrogation of gene functions [9], the elucidation of the regulatory pattern of fetal hemoglobin gene expression [10], etc. Therefore, base editors hold great application prospects in genetic screening by generating DNA sequence diversity. However, the existing base editors can only induce limited DNA sequence diversity due to the limitations of base substrates (a maximum of adenine and cytosine as base substrates at the same time) [11–14] and the constraint to generate only specific single mutations rather than random mutations [15–18]. This potentially hinders their applicability for inducing broader DNA sequence diversity.

In this study, we fused an adenine base editor with an evolved N-methylpurine DNA glycosylase to develop a series of triple base editor-ACG-BEs. ACG-BEs can efficiently catalyze three-base substrates (A, C, or G) and introduce up to nine types of base conversions or transversions (A-to-G/C/T, C-to-T/G/A, and G-to-C/T/A) in HEK293T cells. Moreover, using ACG-BE7, we have identified novel HUDEP-2 monoclonal cell lines harboring distinct A/C/G base substitutions in the *HBG* promoter region, which conferred high expression of γ-globin, suggesting that ACG-BEs are excellent multiple-base-substrate editing tools for genetic screening, gene therapy, or beyond, for inducing more DNA sequence diversity.

## Materials and methods

### Plasmid construction

Primers and plasmid DNA sequences used in this research are listed in the Supplementary Tables 1–4 and Supplementary Sequences 1 and 2 of the Supplementary Information. Human codon-optimized TadA-dual, T_AD_A-3.1, T_AD_A-3.155, MPGv3, and MPG v6.3 were synthesized by Genewiz (Suzhou, China). ABE8e (#138489), BE4max(#112096), AYBE v3(#193967), gGBE v6.3(#202629) and pX330 (#42230) were purchased from Addgene. Polymerase chain reaction (PCR) was performed using KOD-Plus-Neo DNA Polymerase (TOYOBO, Code: KOD-401). Serial plasmids (including ACG-BEs ) generated based on the ABE8e backbone in this article were constructed using ClonExpress MultiS One Step Cloning Kit (Vazyme) (Supplementary Sequences 1 and 2). sgRNA expression plasmids were constructed as described previously [19]. In short, oligonucleotides from Supplementary Table 1 were annealed at 95°C for 5 min, followed by cooling to room temperature, and ligated into BbsI-linearized vectors for sgRNA (Thermo Fisher Scientific).

### Human cell culture and differentiation

HEK293T (ATCC; CRL-3216) and HeLa (ATCC; CCL-2) cell lines were kept in Dulbecco’s Modified Eagle’s medium (Gibco) with 10% (v/v) fetal bovine serum (Gibco) and 1% penicillin–streptomycin (Gibco). HUDEP-2(Δ^G^γ) cells were cultured and expanded in serum-free expansion medium (Stem Cell Technologies) supplemented with human stem cell factor (SCF, 50 ng/ ml, PeproTech), erythropoietin (EPO, 3 IU/ml, PeproTech), dexamethasone (DEX, 1 µM, Sigma), doxycycline (DOX, 1 µg/ml, TAKARA Bio), and 2% penicillin–streptomycin (Gibco). For HUDEP-2 cells differentiation, the cells were induced to differentiate within an EDM (erythroid differentiation media: IMDM, Corning) environment, enriched with a supplement cocktail of including 2% human blood type AB plasma (Sera Care), 1% L-glutamine, 2 IU/ml heparin, 10 µg/ml recombinant human insulin, 3 IU/ml EPO, 330 μg/ml holo-human transferrin (Sigma–Aldrich), 100 ng/ml SCF, 1 µg/ml doxycycline, and 2% penicillin–streptomycin. After 7 days differentiation, the cells were harvested for total RNA preparation. All cell lines used were maintained under standard conditions at 37°C with 5% CO_2_ in the incubator.

### Cell transfection and fluorescence-activated cell sorting

For both DNA on-target and off-target base editing experiments, HEK293T cells were seeded into 24-well plates and transfected at ~80% confluency. Next, a mixture of 3 μl polyethyleneimine (PEI, Polysciences), 1 μg plasmid DNA (750 ng ACG-BEs expression plasmid and 250 ng sgRNA expression plasmid) and serum-free medium was added to the cells. Three days post-transfection, genomic DNA was isolated using the QuickExtract™ DNA Extraction Solution (QE09050, Epicenter) following the manufacturer’s instructions. For an enhanced orthogonal R-loop assay, we utilized an orthogonal R-loop assay for Cas9-independent DNA off-target analysis using a substitution of the dSaCas9-sgRNA plasmid with nSaCas9-sgRNA plasmid at each R-loop site. In the transfection process, a composite of 3 μl polyethyleneimine (PEI, Polysciences), 1 μg plasmid DNA (375 ng nSaCas9-sgRNA plasmid, 375 ng base editor plasmid, and 250 ng sgRNA plasmid) was added to the cells. Following 3 days post-transfection, the transfected cells were digested using 0.25% trypsin (Gibco) for sorting. Thereafter, genomic DNA was extracted using the QuickExtract™ DNA Extraction Solution (QE09050, Epicenter), following the manufacturer’s protocol. For RNA off-target analysis, HEK293T cells were seeded into 10 cm dishes and transfected with 30 μg of nCas9-P2A-GFP, AYBEv3-P2A-GFP, gGBE v6.3-P2A-GFP, TadA-dual(-UGI)-P2A-GFP, and ACG-BEs-P2A-GFP using PEI at ~80% confluency. After 3 days, transfected cells were digested with 0.25% trypsin (Gibco) for fluorescence-activated cell sorting (FACS). FACS was performed on a FACSAria III (BD Biosciences) using FACSDiva version 8.0.2 (BD Biosciences). Cells were gated on their population via forward/sideward scatter after doublet exclusion (Supplementary Note 1). Subsequently, 500000 cells with the top 20% GFP signal were collected, and total mRNA was extracted using RNAiso Plus (Takara). HUDEP-2 cells electroporation was performed using the Celetrix EX + system with 20 μl cuvette strips (Celetrix, Taizhou, China) according to the manufacturer’s instructions. A total of 1 × 10^6^ HUDEP-2 cells were resuspended in 20 μl of electroporation buffer and mixed with 1 μg of plasmid DNA, comprising 750 ng of base editors or CRISPR/Cas9 expression plasmid and 250 ng of sgRNA expression plasmid. Electroporation parameters were conducted at 380 V for 30 ms. After 2 days, FACS was performed on a FACSAria III (BD Biosciences) using FACSDiva version 8.0.2 (BD Biosciences). The Cells were gated on their population via forward/sideward scatter after doublet exclusion (Supplementary Note 2).

### High-throughput DNA sequencing and data analysis

On- and off-target genomic regions were amplified by PCR using primers detailed in Supplementary Tables S2 and S3. High-throughput sequencing (HTS) amplification libraries were prepared by PCR using KOD-Plus-Neo DNA Polymerase and site-specific primers containing an adaptor sequence (forward 5′-ggagtgagtacggtgtgc-3′; Reverse 5′-gagttggatgctggatgg-3′) at their 5′ ends (Supplementary Tables S2 and S3). The resulting products underwent a second PCR using primers containing different barcode sequences. Subsequently, PCR products with different tags were pooled together for deep sequencing on the Illumina HiSeq platform (AnnoroadGene Technology sequenced Co., Ltd). In the sequencing analysis, the reference sequence was carefully curated, commencing 10 base pairs anterior to the protospacer and terminating 10 base pairs posterior to the PAM sequence. The base editing efficiencies (A-to-G/C/T, C-to-T/G/A, and G-to-C/T/A) were quantified using BE-Analyzer [20] or CRISPResso2 [21]. The allele base editing efficiencies (A&G, A&C, C&G, A, C&G) were quantified using the combined BE-Analyzer with a custom script provided in Supplementary Software.

### RNA sequencing experiments

RNA sequencing (RNA-seq) experiments were performed as previously described [22]. Briefly, A total of 3 µg of RNA from each sample was used as input for the RNA sample preparations. Sequencing libraries were generated using the NEBNext Ultra^TM^ RNA Library Prep Kit for Illumina (NEB), following the manufacturer’s guidelines, and subsequently barcoded with unique dual indices using the TruSeq PE Cluster Kit v3-cBot-HS (Illumina). Prior to barcoding, the quality of the libraries was meticulously assessed with the Agilent Bioanalyzer 2100 system. And then, the library preparations were sequenced on an Illumina HiSeq platform and 150 bp paired-end reads were generated.

### RNA sequence variant calling and quality control

RNA sequence variant calling and quality control were performed as previously described [23]. In brief, raw data (raw reads) of fastq format were initially processed through in-house Perl Scripts. First, clean data were obtained by removing adapter-contaminated reads and trimming low-quality bases with Trimmomatic. Meanwhile, we calculated Q20, Q30, and GC content of the clean data. The index of the reference genome was built using Hisat2 v2.0.5, and paired-end clean reads were aligned to the reference genome (Ensemble GRCh38) using Hisat2 v2.0.5. SNP calling was performed using GATK software, version 4.0. Variant loci in base editor overexpression groups were filtered to exclude sites lacking high-confidence reference genotype calls in the control group. The read coverage for a given single-nucleotide variant in the control group should exceed the 90th percentile of the read coverage across all single-nucleotide variants in the corresponding overexpression group. Additionally, these loci were required to have a consensus of at least 99% of reads containing the reference allele in the control experiment. RNA editing events in GFP controls were filtered to include only loci with 10 or more reads and with >0% of reads containing alternate alleles. Base edits were labeled as A-to-I or C-to-U edits based on the following criteria: base edits were classified as A-to-I if identified as A-to-I on the positive strand and corresponding to T-to-C on the negative strand. Similarly, they were classified as C-to-U if identified as C-to-U on the positive strand and corresponding to G-to-A on the negative strand.

### mRNA preparation and quantitative PCR

RNA sequencing experiments were performed as previously described [11]. In brief, 3 μg of total RNA from each sample was used for library preparation. Libraries were generated using the NEBNext Ultra RNA Library Prep Kit for Illumina (NEB) following the manufacturer’s protocol. After quality assessment with the Agilent Bioanalyzer 2100 system, libraries were barcoded with unique dual indices using the TruSeq PE Cluster Kit v3-cBot-HS (Illumina). The resulting RNA-seq libraries were subsequently sequenced on an Illumina HiSeq platform to produce paired-end reads of 150 bp. The isolated mRNA was reverse transcribed using Evo M-MLV RT Mix Kit (Accurate Biology). Quantitative PCR (qPCR) was executed on the QuantiStudio 3 real-time PCR system to ascertain mRNA expression levels. In HUDEP-2(Δ^G^γ) cells, γ-globin mRNA expression levels were calculated as a percentage (γ/γ+β). qPCR primers are listed in Supplementary Table S4.

### Statistics and reproducibility

All statistical analyses were performed on at least *n* = 3 biologically independent experiments using an unpaired two-tailed Student’s t-test or paired two-sided Wilcoxon rank-sum test through Prism software, version 10 (GraphPad). *P* < .05 was considered significant, with specific *P* values are indicated in the figure captions. RNA-seq data were analyzed using Trim Galore (version 0.6.6), STAR (version 2.7.1a), SAMtools (version 1.14), and Picard MarkDuplicates module (version 2.23.9) software. FACS data were analyzed using FlowJo v10.

## Results

### Design and development of triple base editor

**Figure 1.**
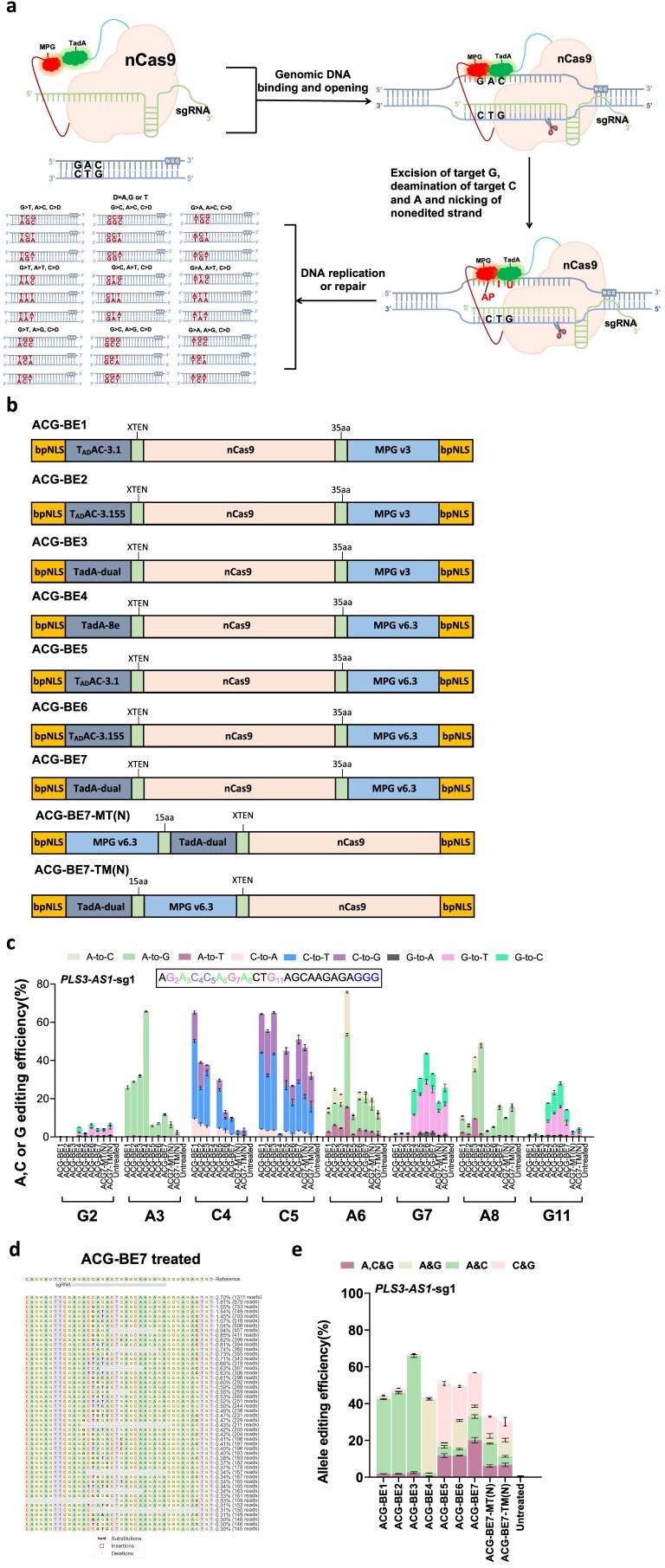
Design and screening of triple base editors (ACG-BEs). (**a**) The process of ACG-BEs catalyzes three types of base substrates (A, C, and G) to generate DNA sequence diversity. Adenosine deaminase refers to evolved *Escherichia coli* adenosine deaminase mutants (TadA-8e, T_AD_AC-3.1, T_AD_AC-3.155, and TadA-dual); nCas9, Cas9 D10A; MPG v6.3, derived from evolved N-methylpurine DNA glycosylase. (**b**) Schematics of the constructs for ACG-BEs. bNLS, bipartite nuclear localization signals; T_AD_AC-3.1, T_AD_AC-3.155, and TadA-dual, derived from evolved *E. coli* adenosine deaminase; nCas9, Cas9 D10A; MPG v3, and MPG v6.3, derived from evolved N-methylpurine DNA glycosylase; linkers are also shown. (**c**) Base-editing outcomes of ACG-BEs at the endogenous target *PLS3-AS1*-sg1 in HEK293T cells. Data are means ± SD (*n* = 3 independent experiments). (**d**) Allele table for *PLS3-AS1*-sg1 in HEK293T cells after ACG-BE7 transfection. The target site allele is boxed in a gray line. The percentile and sequencing read of each allele at one representative of three independent experiments are listed on the right. (**e**) The allele base editing efficiency of ACG-BEs across the protospacer at the endogenous target *PLS3-AS1-*sg1 in HEK293T cells. Data are means ± SD (*n* = 3 independent experiments).

To engineer a triple base editor that can edit three base substrates (A, C, and G), we performed fusion constructs that contained three base substrates editing activity using a fusion of an adenosine deaminase with high cytosine activity, evolved N-methylpurine DNA glycosylase, and nickase Cas9(nCas9) [18, 24, 25] (Fig. 1a). Deaminases are capable of converting adenine to hypoxanthine and cytosine to uracil. Meanwhile, glycosylases can directly excise guanine, as well as the hypoxanthine and uracil that are generated through deamination, thereby creating AP (apurinic/apyrimidinic) sites. Subsequently, these AP sites will undergo random mutations into other bases during the cell’s base excision repair process. Based on this principle, seven triple base editor constructs (named ACG-BE1 to ACG-BE7) were generated(Fig. 1b). These constructs combined three evolved adenosine deaminase mutants (TadA-8e, T_AD_AC-3.1, T_AD_AC-3.155, and TadA-dual), two evolved N-methylpurine DNA glycosylase mutants (MPG v3 and MPG v6.3), and nCas9. A human endogenous target (*PLS3-AS1*-sg1) was then used to test the base editing outcomes of these constructs in HEK293T cells (Fig. 1b). HTS showed that MPG v3-based triple base editors exhibited high A (6.7%–41.7%) and C (37.6%–65.1%) editing efficiency but low G (0.2%–2.1%) editing efficiency (Fig. 1c). However, MPG v6.3-based triple base editors induced A, C, and G editing with high efficiency, ranging from 5.4%–75.7%, 0.3%–51.0%, and 2.1%–43.7%, respectively (Fig. 1c). After further analyzing the two or three bases being simultaneously edited in the same allele, we found that MPG v6.3-based triple base editors induced significantly higher simultaneous A/C/G editing efficiency than corresponding MPG v3-based triple base editors, with ACG-BE7 the most efficient (20.1%) (Fig. 1d and e; Supplementary Fig. S1a). We also selected ACG-BE7 to evaluate the construct orientation of TadA and MPG v6.3 in the MPG v6.3-based triple base editors(Fig. 1b). The results demonstrated that TadA and MPG v6.3 when positioned at the N-terminal and C-terminal of nCas9, respectively, exhibited optimal performance (Fig. 1c and e). Meanwhile, MPG v6.3-based triple base editors also generated A/C, A/G, and C/G simultaneous editing (Fig. 1d and e; Supplementary Fig. S1a). MPG v3-based triple base editors mainly induced A/C simultaneous editing, with ACG-BE3 being the most efficient (63.3%) (Fig. 1e and Supplementary Fig. S1a). Moreover, MPG v6.3-based triple base editors generated more DNA mutant allele types than corresponding MPG v3-based triple base editors (Supplementary Fig. S1b). Therefore, we chose MPG v6.3-based triple base editors (named ACG-BE5–7 henceforward) for further investigation.

### Characterization of triple base editor

**Figure 2.**
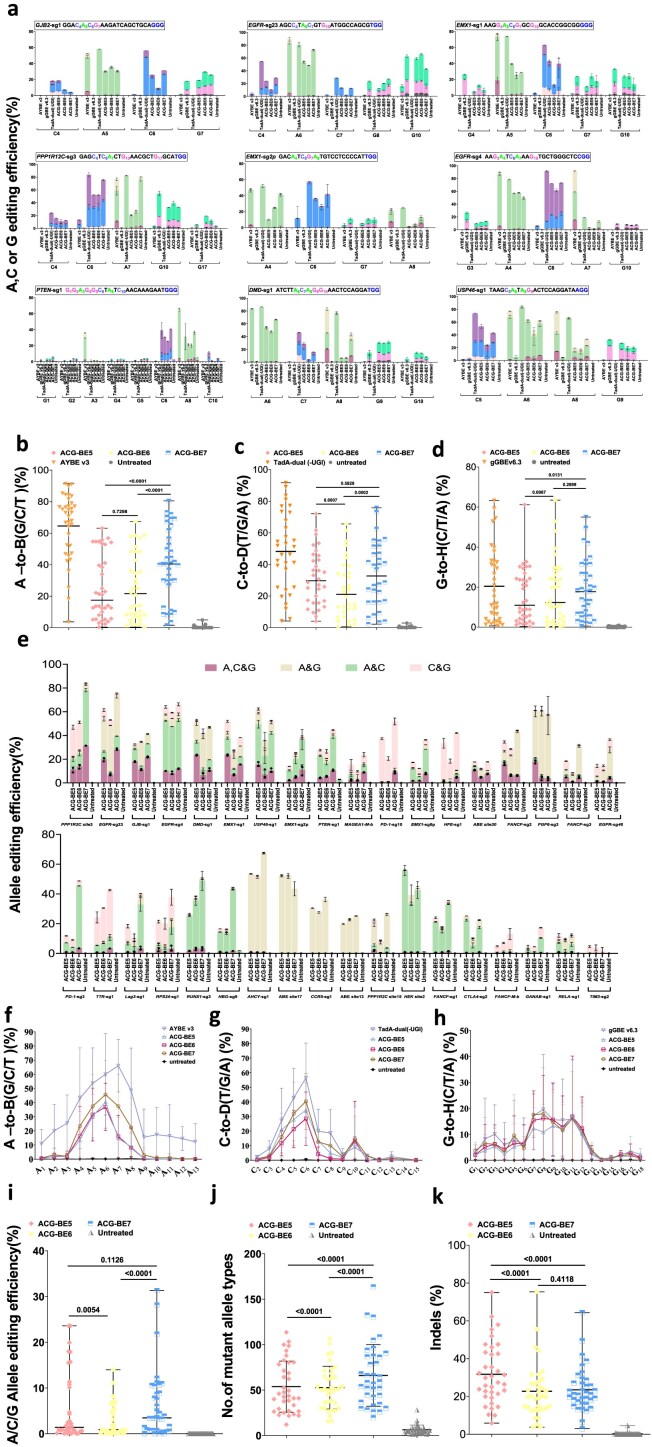
Characteristics of triple base editors (ACG-BEs). (**a**) Base-editing outcome of ACG-BEs at the nine endogenous targets in HEK293T cells. Data are means ± SD (*n* = 3 independent experiments). (**b**) Summary of the A-to-B (G/C/T) editing efficiencies for only the most highly edited adenine induced by ACG-BEs at the 36 target sites in panel (a) and Supplementary Fig. S2. (**c**) Summary of the C-to-D (T/G/A) editing efficiencies for only the most highly edited cytosine induced by ACG-BEs at the 36 target sites in panel (a) and Supplementary Fig. S2. (**d**) Summary of the G-to-H (C/T/A) editing efficiencies for only the most highly edited guanine induced by ACG-BEs at the 36 target sites in panel (a) and Supplementary Fig. S2. (**e**) The allele base editing efficiency of ACG-BEs across protospacer at 36 endogenous target sites in HEK293T cells. Data are means ± SD (*n* = 3 independent experiments). (**f**) Average A-to-B (G/C/T) editing efficiency of ACG-BEs at 36 target sites in panel (a) and Supplementary Fig. S2. Data are means ± SD (*n* = 3 independent experiments). (**g**) Average C-to-D (T/G/A) editing efficiency of ACG-BEs at 36 target sites in panel (a) and Supplementary Fig. S2. Data are means ± SD (*n* = 3 independent experiments). (**h**) Average G-to-H (C/T/A) editing efficiency of ACG-BEs at 36 target sites in panel (a) and Supplementary Fig. S2. Data are means ± SD (*n* = 3 independent experiments). (**i**) Summary of the A/C/G simultaneous editing induced by ACG-BEs at 36 target sites in panel (a) and Supplementary Fig. S2. (**j**) Summary of the number of mutant allele types induced by ACG-BEs at 36 target sites in panel (a) and Supplementary Fig. S2. (**k**) Summary of the indels induced by ACG-BEs at 36 target sites in panel (a) and Supplementary Fig. S2. For (b-d, and i–k), each data point represents means at indicated target sites from three independent experiments. Significance was tested with paired two-sided Wilcoxon rank-sum test (b-d, and i–k).

To unbiasedly profile the characteristic of these optimized triple base editors, 36 endogenous targets containing multiple As, Cs, or Gs were further tested in HEK293T cells (Fig. 2a and Supplementary Fig. S2). After HTS analysis across 36 targets, we found that all triple base editors can act on three bases substrates (As, Cs, and Gs) to perform effective editing on almost all tested targets (Fig. 2a and Supplementary Fig. S2). The A catalytic efficiencies for ACG-BE5, ACG-BE6, and ACG-BE7 were 0.4%–63.1% (median 17.5%), 0.4%–67.4% (median 21.6%), and 1.5%–80.5% (median 40.5%), respectively (Fig. 2a and b and Supplementary Fig. S2). The C catalytic efficiencies for ACG-BE5, ACG-BE6, and ACG-BE7 were 4.0%–72.1% (median 29.7%), 0.6%–65.6% (median 21.1%), and 2.2%–75.8% (median 32.3%) (Fig. 2a and c and Supplementary Fig. S2). The G catalytic efficiencies for ACG-BE5, ACG-BE6, and ACG-BE7 were 0.3%–61.1% (median 11.0%), 0.3%–63.4% (median 12.4%), and 0.5%–55.0% (median 17.7%) (Fig. 2a and d and Supplementary Fig. S2). Notably, the editing activities of ACG-BEs toward bases A, C, and G were all compromised relative to their corresponding single base editors. This reduction in efficiency may be attributed to competitive binding between the adenosine deaminases and N-methylpurine DNA glycosylases to their base substrates (Fig. 2b–d). The major A-to-G/C/T editing windows for ACG-BE5, ACG-BE6, and ACG-BE7 were A_4_–A_8_[counting the end distal to the protospacer-adjacent motif (PAM) as position 1], slightly narrower than that of AYBE v3 (A_3_–A_9_) (Fig. 2a and f and Supplementary Fig. S2; Supplementary Fig. S3a and d). The major C-to-T/G/A editing windows for ACG-BE5, ACG-BE6, and ACG-BE7 were C_4_–C_8_, similar to that of TadA-dual (-UGI) (Fig. 2a and g and Supplementary Fig. S2; Supplementary Fig. S3b and e). The major G-to-T/C/A editing windows for ACG-BE5, ACG-BE6, and ACG-BE7 were G_5_–G_12_, similar to that of gGBE v6.3 (G_3_-G_12_) (Fig. 2a and h and Supplementary Fig. S2; Supplementary Fig. S3c and f). To further analyze the motif preferences of these triple base editors across all tested targets, we found that adenines prone to be edited (>10% on average) by ACG-BE5, ACG-BE6, and ACG-BE7 were located at YAN (Y = C/T, N = A/T/C/G) and GAT. In addition, the AAG motif was preferred by ACG-BE5 and ACG-BE6, but not by ACG-BE7 (Fig. 2a and Supplementary Fig. S2; Supplementary Fig. S4a). Guanines prone to be edited (>10% on average) were located near NGR regions (R = A/G) (Fig. 2a and Supplementary Fig. S2; Supplementary Fig. S4c). But for cytosines, no motif preference was observed (Fig. 2a and Supplementary Fig. S2; Supplementary Fig. S4b). By further analyzing the simultaneous A/C/G editing on the same allele (>1%), ACG-BE5, ACG-BE6, and ACG-BE7 had simultaneous A/C/G editing at 19, 17, and 24 targets of the 36 tested targets, respectively (Fig. 2e). The simultaneous A/C/G editing efficiencies for ACG-BE7 were 1.6%–31.3% (median 3.5%), similar to ACG-BE5(1.1%–23.6%, median 1.4%), which were both higher than that of ACG-BE6(1.1%–14.0%, median 0.9%) (Fig. 2i). Higher simultaneous A/C/G editing efficiencies were observed at targets containing As and Cs at positions 4–8 and Gs at positions 5–12. If the editing windows of A/C/G contained their corresponding preferred motifs, the simultaneous A/C/G editing efficiencies were further improved (Fig. 2a, e, and i and Supplementary Fig. S2). We also compared the simultaneous A/C/G editing efficiencies and DNA mutant types between ACG-BE7 and the mix of AYBE v3, TadA-dual(-UGI), and gGBE v6.3. The results showed that ACG-BE7 exhibited higher simultaneous A/C/G editing efficiencies than that of the mix. And no significant difference for DNA mutant types between the two groups was observed (Supplementary Fig. S5).

Additionally, ACG-BEs can also edit two base substrates (A/C, A/G, C/G) at the same time (>1%) (Fig. 2e). The A/C editing efficiencies for ACG-BE5, ACG-BE6, and ACG-BE7 were 1.7%–55.0% (median 5.1%), 1.1%–39.3% (median 2.2%), and 1.0%–46.9% (median 8.0%), respectively (Fig. 2e). The A/G editing efficiencies for ACG-BE5, ACG-BE6, and ACG-BE7 were 1.0%–52.5% (median 1.4%), 1.0%–55.0% (median 4.9%), and 1.1%–66.5% (median 4.8%), respectively (Fig. 2e). The C/G editing efficiencies for ACG-BE5, ACG-BE6, and ACG-BE7 were 1.2%–36.3% (median 3.8%), 1.2%–23.5% (median 2.6%), and 1.2%–40.3% (median 1.6%), respectively (Fig. 2e). Therefore, ACG-BEs can also be used as dual base editors, such as A&G, C&G, or A&C base editors, at some targets containing only A/C, A/G, and C/G within their editing window. We also compared the spectrum of DNA mutations and indels induced by ACG-BEs, and found that ACG-BE7 induced the most DNA mutations and the lowest rate of indels compared with ACG-BE5 and ACG-BE6 (Fig. 2j and k). Furthermore, efficient A/C/G base-editing outcome for the ACG base editor was also observed in human HeLa cells (Supplementary Fig. S6).

### Off-target evaluation of triple base editor

**Figure 3.**
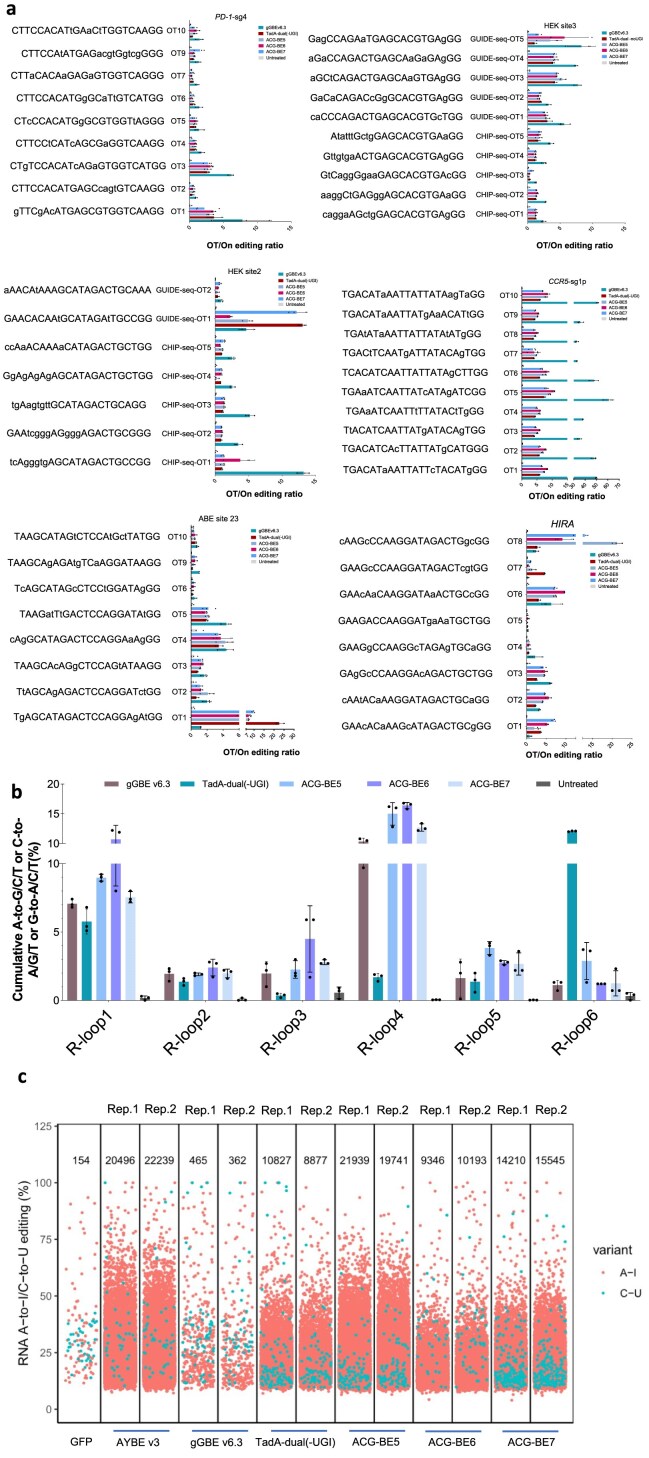
Off-target evaluation of ACG-BEs. (**a**) sgRNA-dependent DNA off- and on-target editing ratios observed at each off-target site by ACG-BEs in HEK293T cells. Lowercase protospacer sequences represent mismatched bases compared to their corresponding on-target sequences. Data are means ± SD (*n* = 3 independent experiments). (**b**) sgRNA-independent DNA off-target base editing induced by ACG-BEs using the modified orthogonal R-loop assay at each R-loop site. Data are means ± SD (*n* = 3 independent experiments). (**c**) Whole-transcriptomic RNA off-target induced by ACG-BEs using RNA-seq. Jitter plots from RNA-seq experiments in HEK293T cells show the efficiencies of C-to-U or A-to-I conversions (*y* axis) with ACG-BEs. Total numbers of modified bases of each treated group are listed at the top.

Next, we performed off-target assessment of ACG-BEs. The results show that compared to corresponding single base editors, ACG-BEs induced comparable or even lower efficiency sgRNA-dependent or sgRNA-independent DNA off-target events by analyzing 52 sgRNA-associated off-target sites or six representative sgRNA-independent random off-target sites (Fig. 3a and b and Supplementary Fig. S7). The RNA-seq analysis showed that ACG-BEs did not exhibit more whole-transcriptomic RNA off-target events compared to AYBE v3, with ACG-BE7 and ACG-BE6 showing 1.5- and 2.2-fold decrease in RNA off-target events, respectively (Fig. 3c). Therefore, ACG-BEs were highly efficient base editing tools with high specificity. Although there were still some off-target events in ACG-BEs, it should be quickly resolved by deaminase protein engineering in the future. Given the efficiency and specificity, we recommend ACG-BE7 and ACG-BE6 to be widely used in the future.

### Saturation editing of A/C/G by **ACG-BEs** in mammalian cells

**Figure 4.**
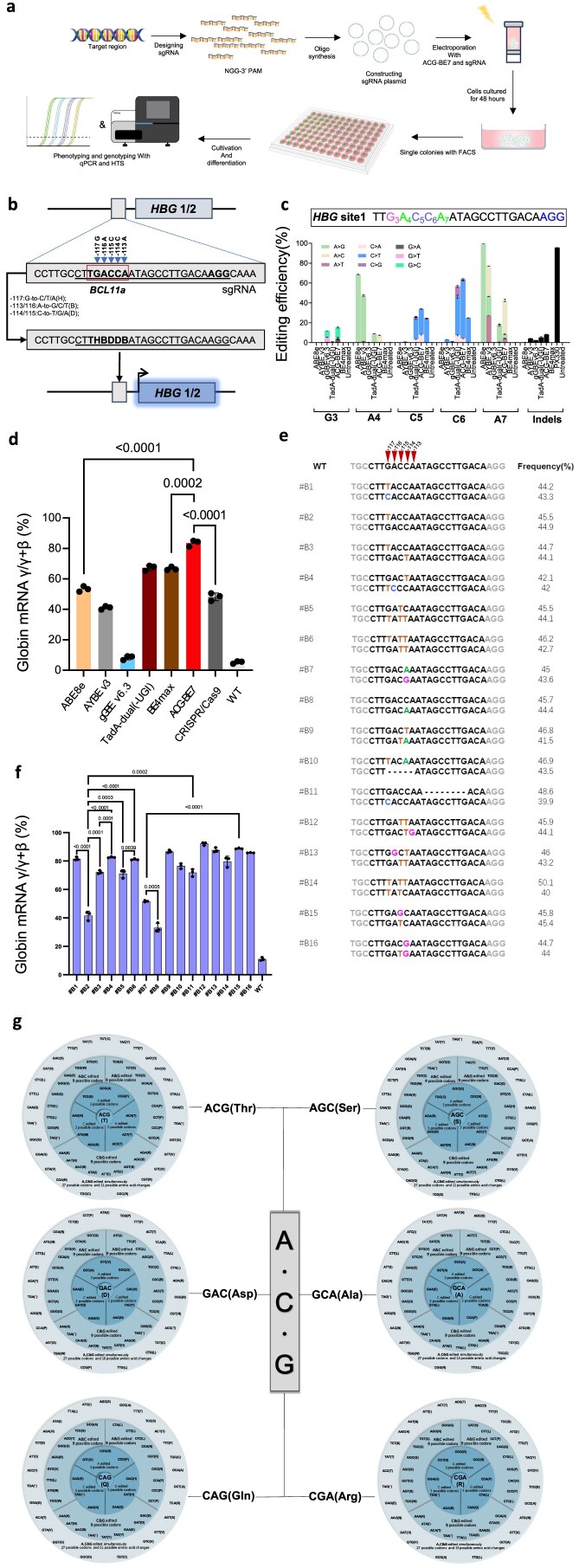
The application of ACG-BEs to induce DNA sequence diversity in coding and non-coding regions of indicated genes in mammalian cells. (**a**) Schematic representation of therapeutic mutations screening with ACG-BEs in HUDEP-2(Δ^G^γ) cells. (**b**) Schematic diagram of ACG-BE7 catalyzes three types of base substrates (−116/113A, −114/115C, and −117G) to generate DNA sequence diversity in the promoter region of *HBG1/2*. The core sequence of the BCL11A binding site is boxed in red. The target sequence is underlined. (**c**) Editing efficiency of ACG-BE7 at *HBG* site 1 in HUDEP-2(Δ^G^γ) cells electroporated with ACG-BE7 and *HBG* site 1. Data are means ± SD (*n* = 3 independent experiments). (**d**) Comparison of γ-globin mRNA expression relative to β-like globin mRNA in HUDEP-2(Δ^G^γ) cells with different gene editors after differentiation. Data are means ± SD (*n* = 3 independent experiments). (**e**) The genotype of HUDEP-2(Δ^G^γ) clone cells generated by ACG-BE7. (**f**) Comparison of γ-globin mRNA expression relative to β-like globin mRNA in HUDEP-2(Δ^G^γ) monoclonal cells with different genotypes after differentiation. Data are means ± SD (*n* = 3 independent experiments). (**g**) Theoretical statistics of codon and amino acid changes for CGA (Arg), CAG (Gln), AGC (Ser), ACG (Thr), GAC (Asp), and GCA (Ala) induced by ACG-BE7 via simultaneous editing of three types of bases. Significance was tested with two-tailed Student’s *t* test (d and f).

To further investigate the possibility of identifying genetic sequence variants with novel functions by inducing DNA sequence diversity using ACG-BEs, we tried to use ACG-BEs to induce new mutations at the promoter of *HBG1/2* region in HUDEP-2(Δ^G^γ) cells, where these mutations would potentially activate the expression of γ-globin and potentially be beneficial for gene therapy of β-hemoglobinopathy (Fig. 4b). We conducted tests in HEK293T cells through co-transfection of ACG-BEs and *HBG* site1 sgRNA, and the results showed that ACG-BE7 can efficiently induce three types of base mutations (Supplementary Fig. S8), indicating that two or three types of A/C/G bases could potentially be edited by ACG-BEs at the promoter regions of the *HBG* site1 in HUDEP-2(Δ^G^γ) cells, thereby enabling the generation of more functional mutants. Next, we further validated their functions by delivering ACG-BE7 along with the sgRNA into HUDEP-2(Δ^G^γ) cells via electroporation (Fig. 4a). After 48 h of electroporation, FACS was performed (Supplementary Note 2), and then the genomic DNA of the cells was collected for HTS. The HTS data showed that all genome-editing tools, including ABE8e, BE4max, and CRISPR/Cas9, could efficiently edit this target in HUDEP-2(Δ^G^γ) cells(Fig.4c). Notably, only TadA-dual or ACG-BE7 can still induce two or three types of base mutations with relatively high efficiency, resulting in two or three types of A/C/G bases being simultaneously edited at the promoter regions of *HBG* site1 in HUDEP-2(Δ^G^γ) cells (Fig. 4c and Supplementary Fig. S9). After 7 days of differentiation, these genome-edited pooled cells exhibited activated γ-globin expression, with ACG-BE7 demonstrating superior γ-globin expression compared to other base editors and CRISPR/Cas9 (Fig. 4d). These data also suggest that ACG-BE7, by targeting the *HBG* promoter region, may become a powerful tool for the future treatment of β-hemoglobinopathies. We further obtained 16 monoclonal cells from ACG-BE7-edited pooled HUDEP-2(Δ^G^γ) cells (Fig. 4e). After 7 days of differentiation, we found that HUDEP-2(Δ^G^γ) monoclonal cell lines carrying different mutations in the *HBG* promoter region could activate the expression of γ-globin to varying degrees (Fig. 4f). Further analysis revealed that the G > T mutation at position −117 (#B2) could effectively activate the expression of γ-globin. However, when both G > T and G > C mutations occurred simultaneously at this position (#B1), the expression of γ-globin was significantly enhanced, reaching 1.9 times the original level (Fig. 4f). This suggests that the contributions of G > T and G > C mutations at position −117 to the activation of γ-globin expression are approximately equally important. The differentiation results of #B2 and #B3 indicated that the introduction of the −114C > T mutation on the basis of the −117G > T mutation (#B3) significantly increased the expression level of γ-globin by 1.7 times, suggesting that the −114C > T mutation has a weaker effect on the expression level of γ-globin than the −117G > T mutation. When the −116A > C mutation was further introduced (#B4), the expression level of γ-globin was further significantly increased, but only by 10.6%, indicating that the −116A > C mutation has a weaker effect on the expression level of γ-globin than the −114C > T mutation (Fig. 4f). The fair comparison of #B2, #B3, and #B4 also indicates that the contributions of the three types of base editing (−117 G > T, −114 C > T, and −116 A > C) to the expression level of γ-globin are stronger than those of the two types of base mutations (−117 G > T, −114 C > T), and all of them are stronger than that of the one type of base mutation (−117 G > T) (Fig. 4f). Both the #B10 and #B11 have indels introduction on the basis of the −117 or −114 mutations, which could further activate the expression of γ-globin (Fig. 4f). This also indicates that the introduction of indels is very important for the expression of γ-globin. Introducing an 8-bp deletion on the basis of the −117 mutation significantly increased the expression of γ-globin (#B11), which was 1.7 times that of #B2, indicating that the introduction of the 8-bp deletion is also very important for the expression of γ-globin, but it is weaker than the −117 mutation (Fig. 4f). Further analysis revealed that #B15, carrying the −115 double allele mutation, had a higher γ-globin expression level than #B7 with the −114 double allele mutation, which also implies that the −115 mutation contributes more to the increase in γ-globin expression level than the −114 mutation (Fig. 4f). Consistent with this conclusion, a comparison of #B2, #B5 and #B6 revealed that the contribution of -115C-to-T to γ-globin expression is also higher than that of -114C-to-T (Fig. 4f). These results demonstrate that the ACG-BEs are a powerful genetic screening tool that can be used to dissect the relationship between base mutations and phenotypes at the base resolution.

Next, we counted DNA codon conversions corresponding to amino acid changes in the gene coding regions induced by ACG-BEs. The results demonstrated that 63 out of 64 DNA codons can be targeted by ACG-BEs, producing 3–63 DNA codon conversions and all 19 amino acids as well as stop codons, can be targeted by ACG-BEs, resulting in 1–19 different amino acid changes (Supplementary Fig. S10 and Supplementary Table S5). Moreover, amino acids Arg (CGA), Gln (CAG), Ser (AGC), Thr (ACG), Asp (GAC), and Ala (GCA), which are composed of A/C/G codons, their three types of bases can be simultaneously edited by ACG-BEs to produce 27 DNA codon conversions and 10–13 amino acid changes (Fig. 4g and Supplementary Table S5). Therefore, ACG-BEs also provide an innovative platform for targeting gene coding regions for genetic screening, molecular evolution, etc.

## Discussion

In this study, we first developed triple base editors by a fusion of an adenosine deaminase with high cytosine activity, evolved N-methylpurine DNA glycosylase, and nCas9. In principle, fusing UGI-free dual base editor and evolved N-methylpurine DNA glycosylase can also be developed into triple base editor system to achieve A-to-G/C/T, C-to-T/G/A, and G-to-C/T/A editing, but its construction is relatively complex with a large size. Therefore, our triple base editors were extremely simplified and compact three-base editing systems that utilized two base substrate (A or C) deaminase activities of TadA-8e and two substrate-excising (I or G) activities of MPG v6.3. The dual function of removing both G and I when editing A/G base substrates by MPG v6.3, derived from gGBE in ACG-BEs, was also a finding in this study. It is the synergistic interaction of these two proteins, TadA-8e and MPG v6.3, that promotes the saturation mutation of the A/G base substrates. However, the detailed mechanism is still unclear, and further research is needed on how these two proteins interact in the future.

Triple base editors can edit not only a single A, C, or G, but also edit A/C/G at the same allele simultaneously. Moreover, compared with the mix of the single base editor, ACG-BEs exhibited higher simultaneous A/C/G editing efficiency, suggesting that ACG-BEs have an advantage in saturation mutagenesis of A/C/G. Additionally, ACG-BEs can function as dual base editors at target sites containing A/C, A/G, or C/G within the editing window, acting as A&C-BEs, A&G-BEs, and C&G-BEs, respectively. ACG-BEs still exhibit detectable off-target activity at both DNA and RNA levels. Further engineering of the TadA domain—such as introducing mutations like V106W, N108Q, M151C, or Q154Y—is expected to substantially reduce these undesired edits in the future. Deaminase-free base editor systems that can edit base T have also been reported [17, 26–28]. Therefore, through further engineering of ACG-BEs and UNG, it is possible to develop a quadruple base editor system or other unreported dual or quadruple base editor systems.

ACG-BEs was used to achieve saturation mutation of three base substrates with one sgRNA targeting specific sites, showing its inherent A/C/G editing advantages, and HUDEP-2 clone cells carrying three base editing substrates with higher γ-globin expression were obtained. Because different mutations (including point mutations and indels) in the *HBG* promoter region have different effects on activating γ-globin expression, this ACG-BE at this site can be regarded as an effective tool for treating β-hemoglobinopathy, which is also a special case. It is worth noting that due to the inherent repair mechanisms of each base of A/C/G, the editing products of A/C/G by ACG-BEs have considerable randomness. More importantly, base editors, including ABEs and CBEs, which can achieve multiple base edits within their editing windows, have also been applied to the screening of oncogenic targets [29] and T cell function [30]. Therefore, ACG-BEs might be another optional and effective tool for genetic screening. If ACG-BEs were further replaced with SpCas9 of PAM-relaxed or near-PAMless SpCas9 variants [31], combining with sgRNA libraries, a better high-throughput screening platform that outperforms existing base editors will be developed.

In summary, we developed triple base editors that can effectively edit three types of base substrates in mammalian cells with high efficiency and specificity, increasing their ability to generate more sequence diversity for broader applications, such as genetic screening, molecular evolution, lineage tracing, and agricultural genetic breeding, etc.

## Supplementary Material

gkaf1423_Supplemental_Files

## Data Availability

Targeted amplicon sequencing data have been deposited in the NCBI Sequence Read Archive Database under Accession Code PRJNA1135466 and PRJNA1163876. The RNA-seq data used in this study have been deposited in the NCBI Sequence Read Archive database under accession code PRJNA1135466. Plasmids encoding ACG-BE5–7 will be available from Addgene (#248890, #248891, and #248892). The custom script to analyze simultaneous A&G, A&C, C&G, A, and C&G editing induced by ACG-BEs can be accessed in the Supplementary Software file, and there are no access restrictions.

## References

[1] Rees HA, Liu DR. Base editing: precision chemistry on the genome and transcriptome of living cells. Nat Rev Genet. 2018;19:770–88. 10.1038/s41576-018-0059-1. 30323312 PMC6535181

[2] Anzalone AV, Koblan LW, Liu DR. Genome editing with CRISPR–Cas nucleases, base editors, transposases and prime editors. Nat Biotechnol. 2020;38:824–44. 10.1038/s41587-020-0561-9. 32572269

[3] Lue NZ, Liau BB. Base editor screens for in situ mutational scanning at scale. Mol Cell. 2023;83:2167–87. 10.1016/j.molcel.2023.06.009. 37390819 PMC10330937

[4] Ma Y, Zhang J, Yin W et al. Targeted AID-mediated mutagenesis (TAM) enables efficient genomic diversification in mammalian cells. Nat Methods. 2016;13:1029–35. 10.1038/nmeth.4027. 27723754

[5] Hess GT, Frésard L, Han K et al. Directed evolution using dCas9-targeted somatic hypermutation in mammalian cells. Nat Methods. 2016;13:1036–42. 10.1038/nmeth.4038. 27798611 PMC5557288

[6] Hanna RE, Hegde M, Fagre CR et al. Massively parallel assessment of human variants with base editor screens. Cell. 2021;184:1064–80. 10.1016/j.cell.2021.01.012. 33606977

[7] Coelho MA, Cooper S, Strauss ME et al. Base editing screens map mutations affecting interferon-γ signaling in cancer. Cancer Cell. 2023;41:288–303. 10.1016/j.ccell.2022.12.009. 36669486 PMC9942875

[8] Walsh ZH, Shah P, Kothapalli N et al. Mapping variant effects on anti-tumor hallmarks of primary human T cells with base-editing screens. Nat Biotechnol. 2024;43:384–95. 10.1038/s41587-024-02235-x. 38783148 PMC12488219

[9] Xu P, Liu Z, Liu Y et al. Genome-wide interrogation of gene functions through base editor screens empowered by barcoded sgRNAs. Nat Biotechnol. 2021;39:1403–13. 10.1038/s41587-021-00944-1. 34155407

[10] Cheng L, Li Y, Qi Q et al. Single-nucleotide-level mapping of DNA regulatory elements that control fetal hemoglobin expression. Nat Genet. 2021;53:869–80. 10.1038/s41588-021-00861-8. 33958780 PMC8628368

[11] Zhang X, Zhu B, Chen L et al. Dual base editor catalyzes both cytosine and adenine base conversions in human cells. Nat Biotechnol. 2020;38:856–60. 10.1038/s41587-020-0527-y. 32483363

[12] Sakata RC, Ishiguro S, Mori H et al. Base editors for simultaneous introduction of C-to-T and A-to-G mutations. Nat Biotechnol. 2020;38:865–9. 10.1038/s41587-020-0509-0. 32483365

[13] Li C, Zhang R, Meng X et al. Targeted, random mutagenesis of plant genes with dual cytosine and adenine base editors. Nat Biotechnol. 2020;38:875–82. 10.1038/s41587-019-0393-7. 31932727

[14] Grünewald J, Zhou R, Lareau CA et al. A dual-deaminase CRISPR base editor enables concurrent adenine and cytosine editing. Nat Biotechnol. 2020;38:861–4. 10.1038/s41587-020-0535-y. 32483364 PMC7723518

[15] Koblan LW, Arbab M, Shen MW et al. Efficient C• G-to-G• C base editors developed using CRISPRi screens, target-library analysis, and machine learning. Nat Biotechnol. 2021;39:1414–25. 10.1038/s41587-021-00938-z. 34183861 PMC8985520

[16] Chen L, Hong M, Luan C et al. Adenine transversion editors enable precise, efficient A• T-to-C• G base editing in mammalian cells and embryos. Nat Biotechnol. 2024;42:638–50. 10.1038/s41587-023-01821-9. 37322276

[17] Ye L, Zhao D, Li J et al. Glycosylase-based base editors for efficient T-to-G and C-to-G editing in mammalian cells. Nat Biotechnol. 2024;42:1538–47. 38168994 10.1038/s41587-023-02050-w

[18] Tong H, Liu N, Wei Y et al. Programmable deaminase-free base editors for G-to-Y conversion by engineered glycosylase. Natl Sci Rev. 2023;10:nwad143. 10.1093/nsr/nwad143. 37404457 PMC10317176

[19] Yang L, Zhang X, Wang L et al. Increasing targeting scope of adenosine base editors in mouse and rat embryos through fusion of TadA deaminase with Cas9 variants. Protein Cell. 2018;9:814–9. 10.1007/s13238-018-0568-x. 30066232 PMC6107483

[20] Hwang GH, Park J, Lim K et al. Web-based design and analysis tools for CRISPR base editing. BMC Bioinformatics. 2018;19:542. 10.1186/s12859-018-2585-4. 30587106 PMC6307267

[21] Clement K, Rees H, Canver MC et al. CRISPResso2 provides accurate and rapid genome editing sequence analysis. Nat Biotechnol. 2019;37:224–6. 10.1038/s41587-019-0032-3. 30809026 PMC6533916

[22] Zhang X, Chen L, Zhu B et al. Increasing the efficiency and targeting range of cytidine base editors through fusion of a single-stranded DNA-binding protein domain. Nat Cell Biol. 2020;22:740–50. 10.1038/s41556-020-0518-8. 32393889

[23] Zhou C, Sun Y, Yan R et al. Off-target RNA mutation induced by DNA base editing and its elimination by mutagenesis. Nature. 2019;571:275–8. 10.1038/s41586-019-1314-0. 31181567

[24] Neugebauer ME, Hsu A, Arbab M et al. Evolution of an adenine base editor into a small, efficient cytosine base editor with low off-target activity. Nat Biotechnol. 2023;41:673–85. 10.1038/s41587-022-01533-6. 36357719 PMC10188366

[25] Lam DK, Feliciano PR, Arif A et al. Improved cytosine base editors generated from TadA variants. Nat Biotechnol. 2023;41:686–97. 10.1038/s41587-022-01611-9. 36624149 PMC10188367

[26] Yi Z, Zhang X, Wei X et al. Programmable DNA pyrimidine base editing via engineered uracil-DNA glycosylase. Nat Commun. 2024;15:6397. 10.1038/s41467-024-50012-w. 39080265 PMC11289083

[27] Tong H, Wang H, Wang X et al. Development of deaminase-free T-to-S base editor and C-to-G base editor by engineered human uracil DNA glycosylase. Nat Commun. 2024;15:4897. 10.1038/s41467-024-49343-5. 38851742 PMC11162499

[28] He Y, Zhou X, Chang C et al. Protein language models-assisted optimization of a uracil-N-glycosylase variant enables programmable T-to-G and T-to-C base editing. Mol Cell. 2024;84:1257–70. 10.1016/j.molcel.2024.01.021. 38377993

[29] Coelho MA, Cooper S, Strauss ME et al. Base editing screens map mutations affecting interferon-gamma signaling in cancer. Cancer Cell. 2023;41:288–303. 10.1016/j.ccell.2022.12.009. 36669486 PMC9942875

[30] Walsh ZH, Shah P, Kothapalli N et al. Mapping variant effects on anti-tumor hallmarks of primary human T cells with base-editing screens. Nat Biotechnol. 2025;43:384–95. 10.1038/s41587-024-02235-x. 38783148 PMC12488219

[31] Walton RT, Christie KA, Whittaker MN et al. Unconstrained genome targeting with near-PAMless engineered CRISPR-Cas9 variants. Science. 2020;368:290–6. 10.1126/science.aba8853. 32217751 PMC7297043

