# Supplementary material for: Triple base editor catalyzes saturation mutation of adenine, cytidine, and guanine
Source: Nucleic Acids Res. 2026 Jan 14;54(2):gkaf1423. doi: 10.1093/nar/gkaf1423 (PMC12802905; doi:10.1093/nar/gkaf1423)
Supplement: gkaf1423_Supplemental_Files [file gkaf1423_supplemental_files.zip › Supplementary Sequences 251120.pdf]

Supplementary Sequences

Supplementary Sequences 1 . DNA sequences used this study for cell transfection. Within base editor sequences, bp-NLS sequences are in red, TadA are in brown, linkers are in yellow, Cas9 nickase sequence is in bright green, and MPG sequences are in grey.

ACG-BE1

ATGAAACGGACAGCCGACGGAAGCGAGTTCGAGTCACCAAAGAAGAAGCGGAAAGTCTCTGAGGTGGAGTTTTCCCACGAGTACTGGA
TGAGACATGCCCTGACCCTGGCCAAGAGGGCACGGGATGAGAGGAGCGTGCCTGTGGGAGCCGTGCTGGTGCTGAACAATAGAGTGAT
CGGCGAGGGCTGGAACAGAGCCAAGGGCCTGCACGACCCAACAGCCCATGCCGAAATTATGGCCCTGAGACAGGGCGGCCTGGTCATG
CAGAACTACAGACTGTACGACGCCACCCTGTACACCACATTCGAGCCTTGCGTGATGTGCGCCGGCGCCATGATCCACTCTAGGATCGGCC
GCGTGGTGTTTGGCGTGAGGAACGCCAAAACCGGCGCCGCAGGCTCCCTGATGGACGTGCTGCACCACCCCGGCATGAATCACCGCGTC
GAAATTACCGAGGGAATCCTGGCAGATGAATGTGCCGCCCTGCTGTGCAGGTTCTTCCGGATGCCTAGAAGGGTGTTCAATGCTCAGAAG
AAGGCCCAGAGCTCCACCGACTCCGGAGGATCTAGCGGAGGCTCCTCTGGCTCTGAGACACCTGGCACAAGCGAGAGCGCAACACCTG
AAAGCAGCGGGGGCAGCAGCGGGGGGTCAGATAAAAAGTATTCTATTGGTTTAGCCATCGGCACTAATTCCGTTGGATGGGCTGTCATAA
CCGATGAATACAAAGTACCTTCAAAGAAATTTAAGGTGTTGGGGAACACAGACCGTCATTCGATTAAAAAGAATCTTATCGGTGCCCTCCT
ATTCGATAGTGGCGAAACGGCAGAGGCGACTCGCCTGAAACGAACCGCTCGGAGAAGGTATACACGTCGCAAGAACCGAATATGTTACT
TACAAGAAATTTTTAGCAATGAGATGGCCAAAGTTGACGATTCTTTCTTTCACCGTTTGGAAGAGTCCTTCCTTGTCGAAGAGGACAAGA
AACATGAACGGCACCCCATCTTTGGAAACATAGTAGATGAGGTGGCATATCATGAAAAGTACCCAACGATTTATCACCTCAGAAAAAAGCT
AGTTGACTCAACTGATAAAGCGGACCTGAGGTTAATCTACTTGGCTCTTGCCCATATGATAAAGTTCCGTGGGCACTTTCTCATTGAGGGTG
ATCTAAATCCGGACAACTCGGATGTCGACAAACTGTTCATCCAGTTAGTACAAACCTATAATCAGTTGTTTGAAGAGAACCCTATAAATGCA
AGTGGCGTGGATGCGAAGGCTATTCTTAGCGCCCGCCTCTCTAAATCCCGACGGCTAGAAAACCTGATCGCACAATTACCCGGAGAGAAG
AAAAATGGGTTGTTCGGTAACCTTATAGCGCTCTCACTAGGCCTGACACCAAATTTTAAGTCGAACTTCGACTTAGCTGAAGATGCCAAATT
GCAGCTTAGTAAGGACACGTACGATGACGATCTCGACAATCTACTGGCACAAATTGGAGATCAGTATGCGGACTTATTTTTGGCTGCCAAA
AACCTTAGCGATGCAATCCTCCTATCTGACATACTGAGAGTTAATACTGAGATTACCAAGGCGCCGTTATCCGCTTCAATGATCAAAAGGTAC
GATGAACATCACCAAGACTTGACACTTCTCAAGGCCCTAGTCCGTCAGCAACTGCCTGAGAAATATAAGGAAATATTCTTTGATCAGTCGA
AAAACGGGTACGCAGGTTATATTGACGGCGGAGCGAGTCAAGAGGAATTCTACAAGTTTATCAAACCCATATTAGAGAAGATGGATGGGA
CGGAAGAGTTGCTTGTAAAACTCAATCGCGAAGATCTACTGCGAAAGCAGCGGACTTTCGACAACGGTAGCATTCCACATCAAATCCACT
TAGGCGAATTGCATGCTATACTTAGAAGGCAGGAGGATTTTTATCCGTTCCTCAAAGACAATCGTGAAAAGATTGAGAAAATCCTAACCTT
TCGCATACCTTACTATGTGGGACCCCTGGCCCGAGGGAACTCTCGGTTCGCATGGATGACAAGAAAGTCCGAAGAAACGATTACTCCATG
GAATTTTGAGGAAGTTGTCGATAAAGGTGCGTCAGCTCAATCGTTCATCGAGAGGATGACCAACTTTGACAAGAATTTACCGAACGAAAA
AGTATTGCCTAAGCACAGTTTACTTTACGAGTATTTCACAGTGTACAATGAACTCACGAAAGTTAAGTATGTCACTGAGGGCATGCGTAAAC
CCGCCTTTCTAAGCGGAGAACAGAAGAAAGCAATAGTAGATCTGTTATTCAAGACCAACCGCAAAGTGACAGTTAAGCAATTGAAAGAG
GACTACTTTAAGAAAATTGAATGCTTCGATTCTGTCGAGATCTCCGGGGTAGAAGATCGATTTAATGCGTCACTTGGTACGTATCATGACCT
CCTAAAGATAATTAAAGATAAGGACTTCCTGGATAACGAAGAGAATGAAGATATCTTAGAAGATATAGTGTTGACTCTTACCCTCTTTGAAG
ATCGGGAAATGATTGAGGAAAGACTAAAAACATACGCTCACCTGTTCGACGATAAGGTTATGAAACAGTTAAAGAGGCGTCGCTATACGG
GCTGGGGACGATTGTCGCGGAAACTTATCAACGGGATAAGAGACAAGCAAAGTGGTAAAACTATTCTCGATTTTCTAAAGAGCGACGGCT
TCGCCAATAGGAACTTTATGCAGCTGATCCATGATGACTCTTTAACCTTCAAAGAGGATATACAAAAGGCACAGGTTTCCGGACAAGGGGA
CTCATTGCACGAACATATTGCGAATCTTGCTGGTTCGCCAGCCATCAAAAAGGGCATACTCCAGACAGTCAAAGTAGTGGATGAGCTAGTT
AAGGTCATGGGACGTCACAAACCGGAAAACATTGTAATCGAGATGGCACGCGAAAATCAAACGACTCAGAAGGGGCAAAAAAACAGTC
GAGAGCGGATGAAGAGAATAGAAGAGGGTATTAAAGAACTGGGCAGCCAGATCTTAAAGGAGCATCCTGTGGAAAATACCCAATTGCAG
AACGAGAAACTTTACCTCTATTACCTACAAAATGGAAGGGACATGTATGTTGATCAGGAACTGGACATAAACCGTTTATCTGATTACGACGT
CGATCACATTGTACCCCAATCCTTTTTGAAGGACGATTCAATCGACAATAAAGTGCTTACACGCTCGGATAAGAACCGAGGGAAAAGTGAC
AATGTTCCAAGCGAGGAAGTCGTAAAGAAAATGAAGAACTATTGGCGGCAGCTCCTAAATGCGAAACTGATAACGCAAAGAAAGTTCGA
TAACTTAACTAAAGCTGAGAGGGGTGGCTTGTCTGAACTTGACAAGGCCGGATTTATTAAACGTCAGCTCGTGGAAACCCGCCAAATCAC
AAAGCATGTTGCACAGATACTAGATTCCCGAATGAATACGAAATACGACGAGAACGATAAGCTGATTCGGGAAGTCAAAGTAATCACTTTA
AAGTCAAAATTGGTGTCGGACTTCAGAAAGGATTTTCAATTCTATAAAGTTAGGGAGATAAATAACTACCACCATGCGCACGACGCTTATCT
TAATGCCGTCGTAGGGACCGCACTCATTAAGAAATACCCGAAGCTAGAAAGTGAGTTTGTGTATGGTGATTACAAAGTTTATGACGTCCGT

AAGATGATCGCGAAAAGCGAACAGGAGATAGGCAAGGCTACAGCCAAATACTTCTTTTATTCTAACATTATGAATTTCTTTAAGACGGAAA
TCACTCTGGCAAACGGAGAGATACGCAAACGACCTTTAATTGAAACCAATGGGGAGACAGGTGAAATCGTATGGGATAAGGGCCGGGAC
TTCGCGACGGTGAGAAAAGTTTTGTCCATGCCCCAAGTCAACATAGTAAAGAAAACTGAGGTGCAGACCGGAGGGTTTTCAAAGGAATC
GATTCTTCCAAAAAGGAATAGTGATAAGCTCATCGCTCGTAAAAAGGACTGGGACCCGAAAAAGTACGGTGGCTTCGATAGCCCTACAGT
TGCCTATTCTGTCCTAGTAGTGGCAAAAGTTGAGAAGGGAAAATCCAAGAAACTGAAGTCAGTCAAAGAATTATTGGGGATAACGATTAT
GGAGCGCTCGTCTTTTGAAAAGAACCCCATCGACTTCCTTGAGGCGAAAGGTTACAAGGAAGTAAAAAAGGATCTCATAATTAAACTACC
AAAGTATAGTCTGTTTGAGTTAGAAAATGGCCGAAAACGGATGTTGGCTAGCGCCGGAGAGCTTCAAAAGGGGAACGAACTCGCACTAC
CGTCTAAATACGTGAATTTCCTGTATTTAGCGTCCCATTACGAGAAGTTGAAAGGTTCACCTGAAGATAACGAACAGAAGCAACTTTTTGTT
GAGCAGCACAAACATTATCTCGACGAAATCATAGAGCAAATTTCGGAATTCAGTAAGAGAGTCATCCTAGCTGATGCCAATCTGGACAAAG
TATTAAGCGCATACAACAAGCACAGGGATAAACCCATACGTGAGCAGGCGGAAAATATTATCCATTTGTTTACTCTTACCAACCTCGGCGCT
CCAGCCGCATTCAAGTATTTTGACACAACGATAGATCGCAAACGATACACTTCTACCAAGGAGGTGCTAGACGCGACACTGATTCACCAAT
CCATCACGGGATTATATGAAACTCGGATAGATTTGTCACAGCTTGGGGGTGACAGCGGCGGCTCCAAAAGAACCGCCGACGGCAGCGAA
TTCGAGCCCAAGAAGAAACGGAAGGTGCTGGGCGGCGACAGCGGGGGGTCCGGGGGCTCCGGCGGGAGCGTGACCCCCGCCCTGCA
GATGAAGAAGCCCAAGCAGTTCTGCAGAAGAATGGGGCAGAAGAAGCAGAGACCTGCTCGGGCTGGGCAGCCTCACAGCAGCAGCGA
CGCCGCCCAAGCTCCTGCCGAGCAGCCTCATAGCAGCAGCGACGCTGCTCAAGCCCCTTGCCCTAGAGAGCGGTGCCTGGGGCCTCCCA
CCACCCCCGGCCCCTACAGAAGCATCTACTTCAGCAGCCCCAAGGGCCACCTGACAAGACTGGGCCTGGAGTTCTTCGATCAGCCCGCCG
TGCCCCTGGCTAGAGCCTTCCTGGGCCAAGTGCTGGTGAGAAGACTGCCCAACGGCACCGAGCTGAGAGGCAGAATCGTGGAGACCGA
GGCCTACCTGGGCCCCGAGGACGAAGCTGCCCACAGCAGAGGCGGCAGACAGACCCCTAGAAACAGAGGCATGTTCATGAAGCCCGGC
ACCCTGTACGTGTACATCATCTACAGAATGTACTTCTGCATGTCCATCAGCAGCCAAGGCGACGGCGCCTGCGTGCTGCTCAGAGCCCTGG
AGCCCCTGGAGGGCCTGGAGACCATGAGACAGCTGAGAGCCACCCTGAGAGCTGCCACCGCCGCTAGAGTGCTCGCCGACAGAGAGCT
GTGCAGCGGCCCTAGCAAGCTGTGCCAAGCCCTGGCCATCAACAAGAGCTTCGATCAGAGAGACCTGGCCCAAGACGAGGCCGTGTGG
CTGGAGAGAGGCCCTCTGGAGCCTAGCGAACCCGCTGTGGTGGCCGCTGCCCGGGTGGGGGTCGGGCACGCCGGCGAGTGGGCTAGA
AAGCCCCTGAGATTCTACGTGAGAGGCAGCCCCTGGGTGAGCGTGGTGGACAGAGTCGCCGAGCAAGACACCCAAGCCTCCGGCGGCA
GCAAGAGAACAGCCGACGGCTCCGAGTTTGAACCCAAGAAAAAGAGAAAAGTGTAA

ACG-BE2

ATGAAACGGACAGCCGACGGAAGCGAGTTCGAGTCACCAAAGAAGAAGCGGAAAGTCTCTGAGGTGGAGTTTTCCCACGAGTACTGGA
TGAGACATGCCCTGACCCTGGCCAAGAGGGCACGGGATGAGAGGAGCGTGCCTGTGGGAGCCGTGCTGGTGCTGAACAATAGAGTGAT
CGGCGAGGGCTGGAACAGAGCCATCGGCCTGCACGACCCAACAGCCCATGCCGAAATTATGGCCCTGAGACAGGGCGGCCTGGTCATG
CAGAACTACAGACTGTACGACGCCACCCTGTACACCACATTCGAGCCTTGCGTGATGTGCGCCGGCGCCATGATCCACTCTAGGATCGGCC
GCGTGGTGTTTGGCGTGAGGAACGCCAAAACCGGCGCCGCAGGCTCCCTGATGGACGTGCTGCACCACCCCGGCATGAATCACCGCGTC
GAAATTACCGAGGGAATCCTGGCAGATGAATGTGCCGCCCTGCTGTGCAGGTTCTTCCGGATGCCTAGAAGGGTGTTCAATGCTCAGAAG
AAGGCCCAGAGCTCCACCGACTCCGGAGGATCTAGCGGAGGCTCCTCTGGCTCTGAGACACCTGGCACAAGCGAGAGCGCAACACCTG
AAAGCAGCGGGGGCAGCAGCGGGGGGTCA___Cas9n___AGCGGCGGCTCCAAAAGAACCGCCGACGGCAGCGAATTCGAGCCCAA
GAAGAAACGGAAGGTGCTGGGCGGCGACAGCGGGGGGTCCGGGGGCTCCGGCGGGAGCGTGACCCCCGCCCTGCAGATGAAGAAGC
CCAAGCAGTTCTGCAGAAGAATGGGGCAGAAGAAGCAGAGACCTGCTCGGGCTGGGCAGCCTCACAGCAGCAGCGACGCCGCCCAAG
CTCCTGCCGAGCAGCCTCATAGCAGCAGCGACGCTGCTCAAGCCCCTTGCCCTAGAGAGCGGTGCCTGGGGCCTCCCACCACCCCCGGC
CCCTACAGAAGCATCTACTTCAGCAGCCCCAAGGGCCACCTGACAAGACTGGGCCTGGAGTTCTTCGATCAGCCCGCCGTGCCCCTGGCT
AGAGCCTTCCTGGGCCAAGTGCTGGTGAGAAGACTGCCCAACGGCACCGAGCTGAGAGGCAGAATCGTGGAGACCGAGGCCTACCTGG
GCCCCGAGGACGAAGCTGCCCACAGCAGAGGCGGCAGACAGACCCCTAGAAACAGAGGCATGTTCATGAAGCCCGGCACCCTGTACGT
GTACATCATCTACAGAATGTACTTCTGCATGTCCATCAGCAGCCAAGGCGACGGCGCCTGCGTGCTGCTCAGAGCCCTGGAGCCCCTGGA
GGGCCTGGAGACCATGAGACAGCTGAGAGCCACCCTGAGAGCTGCCACCGCCGCTAGAGTGCTCGCCGACAGAGAGCTGTGCAGCGGC
CCTAGCAAGCTGTGCCAAGCCCTGGCCATCAACAAGAGCTTCGATCAGAGAGACCTGGCCCAAGACGAGGCCGTGTGGCTGGAGAGAG
GCCCTCTGGAGCCTAGCGAACCCGCTGTGGTGGCCGCTGCCCGGGTGGGGGTCGGGCACGCCGGCGAGTGGGCTAGAAAGCCCCTGA
GATTCTACGTGAGAGGCAGCCCCTGGGTGAGCGTGGTGGACAGAGTCGCCGAGCAAGACACCCAAGCCTCCGGCGGCAGCAAGAGAA
CAGCCGACGGCTCCGAGTTTGAACCCAAGAAAAAGAGAAAAGTGTAA

ACG-BE3

ATGAAACGGACAGCCGACGGAAGCGAGTTCGAGTCACCAAAGAAGAAGCGGAAAGTCTCTGAGGTGGAGTTTTCCCACGAGTACTGGA
TGAGACATGCCCTGACCCTGGCCAAGAGGGCACGGGATGAGGGCGAGGCCCCTGTGGGAGCCGTGCTGGTGCTGAACAATAGAGTGAT
CGGCGAGGGCTGGAACAGAAGAATCGGCCTGCACGACCCAACAGCCCATGCCGAAATTATGGCCCTGAGACAGGGCGGCCTGGTCATG
CAGAACAGCAGACTGATTGACGCCACCCTGTACGTGACATTCGAGCCTTGCGTGATGTGCGCCGGCGCCATGATCAACTCTAGGATCGGC
CGCGTGGTGTTTGGCGTGAGGAACTCAAAAAGAGGCGCCGCAGGCTCCCTGATGAACGTGCTGAACTACCCCGGCATGAATCACCGCGT
CGAAATTACCGAGGGAATCCTGGCAGATGAATGTGCCGCCCTGCTGTGCGATTTCTATCGGATGCCTAGACAGGTGTTCAATGCTCAGAAG
AAGGCCCAGAGCTCCATCAACTCCGGAGGATCTAGCGGAGGCTCCTCTGGCTCTGAGACACCTGGCACAAGCGAGAGCGCAACACCTGA
AAGCAGCGGGGGCAGCAGCGGGGGGTCA___Cas9n___AGCGGCGGCTCCAAAAGAACCGCCGACGGCAGCGAATTCGAGCCCAAG
AAGAAACGGAAGGTGCTGGGCGGCGACAGCGGGGGGTCCGGGGGCTCCGGCGGGAGCGTGACCCCCGCCCTGCAGATGAAGAAGCC
CAAGCAGTTCTGCAGAAGAATGGGGCAGAAGAAGCAGAGACCTGCTCGGGCTGGGCAGCCTCACAGCAGCAGCGACGCCGCCCAAGC
TCCTGCCGAGCAGCCTCATAGCAGCAGCGACGCTGCTCAAGCCCCTTGCCCTAGAGAGCGGTGCCTGGGGCCTCCCACCACCCCCGGCC
CCTACAGAAGCATCTACTTCAGCAGCCCCAAGGGCCACCTGACAAGACTGGGCCTGGAGTTCTTCGATCAGCCCGCCGTGCCCCTGGCTA
GAGCCTTCCTGGGCCAAGTGCTGGTGAGAAGACTGCCCAACGGCACCGAGCTGAGAGGCAGAATCGTGGAGACCGAGGCCTACCTGG
GCCCCGAGGACGAAGCTGCCCACAGCAGAGGCGGCAGACAGACCCCTAGAAACAGAGGCATGTTCATGAAGCCCGGCACCCTGTACGT
GTACATCATCTACAGAATGTACTTCTGCATGTCCATCAGCAGCCAAGGCGACGGCGCCTGCGTGCTGCTCAGAGCCCTGGAGCCCCTGGA
GGGCCTGGAGACCATGAGACAGCTGAGAGCCACCCTGAGAGCTGCCACCGCCGCTAGAGTGCTCGCCGACAGAGAGCTGTGCAGCGGC
CCTAGCAAGCTGTGCCAAGCCCTGGCCATCAACAAGAGCTTCGATCAGAGAGACCTGGCCCAAGACGAGGCCGTGTGGCTGGAGAGAG
GCCCTCTGGAGCCTAGCGAACCCGCTGTGGTGGCCGCTGCCCGGGTGGGGGTCGGGCACGCCGGCGAGTGGGCTAGAAAGCCCCTGA
GATTCTACGTGAGAGGCAGCCCCTGGGTGAGCGTGGTGGACAGAGTCGCCGAGCAAGACACCCAAGCCTCCGGCGGCAGCAAGAGAA
CAGCCGACGGCTCCGAGTTTGAACCCAAGAAAAAGAGAAAAGTGTAA

ACG-BE4

ATGAAACGGACAGCCGACGGAAGCGAGTTCGAGTCACCAAAGAAGAAGCGGAAAGTCTCTGAGGTGGAGTTTTCCCACGAGTACTGGA
TGAGACATGCCCTGACCCTGGCCAAGAGGGCACGGGATGAGAGGGAGGTGCCTGTGGGAGCCGTGCTGGTGCTGAACAATAGAGTGAT
CGGCGAGGGCTGGAACAGAGCCATCGGCCTGCACGACCCAACAGCCCATGCCGAAATTATGGCCCTGAGACAGGGCGGCCTGGTCATG
CAGAACTACAGACTGATTGACGCCACCCTGTACGTGACATTCGAGCCTTGCGTGATGTGCGCCGGCGCCATGATCCACTCTAGGATCGGCC
GCGTGGTGTTTGGCGTGAGGAACTCAAAAAGAGGCGCCGCAGGCTCCCTGATGAACGTGCTGAACTACCCCGGCATGAATCACCGCGTC
GAAATTACCGAGGGAATCCTGGCAGATGAATGTGCCGCCCTGCTGTGCGATTTCTATCGGATGCCTAGACAGGTGTTCAATGCTCAGAAGA
AGGCCCAGAGCTCCATCAACTCCGGAGGATCTAGCGGAGGCTCCTCTGGCTCTGAGACACCTGGCACAAGCGAGAGCGCAACACCTGAA
AGCAGCGGGGGCAGCAGCGGGGGGTCA___Cas9n___AGCGGCGGCTCCAAAAGAACCGCCGACGGCAGCGAATTCGAGCCCAAGA
AGAAACGGAAGGTGCTGGGCGGCGACAGCGGGGGGTCCGGGGGCTCCGGCGGGAGCGTGACCCCCGCCCTGCAGATGAAGAAGCCC
AAGCAGTTCTGCAGAAGAATGGGGCAGAAGAAGCAGAGACCTGCTCGGGCTGGGCAGCCTCACAGCAGCAGCGACGCCGCCCAAGCT
CCTGCCGAGCAGCCTCATAGCAGCAGCGACGCTGCTCAAGCCCCTTGCCCTAGAGAGCGGTGCCTGGGGCCTCCCACCACCCCCGGCCC
CTACAGAAGCATCTACTTCAGCAGCCCCAAGGGCCACCTGACAAGACTGGGCCTGGAGTTCTTCGATCAGCCCGCCGTGCCCCTGGCTAG
AGCCTTCCTGGGCCAAGTGCTGGTGAGAAGACTGCCCAACGGCACCGAGCTGAGAGGCAGAATCGTGGAGACCGAGGCCTACCTGGGC
CCCGAGGACGAAGCTGCCCACAGCAGAGGCGGCAGACAGACCCCTAGAAACAGAGGCATGTTCATGAAGCCCGGCACCCTGTACGTGT
ACATCATCTACAGAATGTACTTCTGCATGGGCATCAGCAGCCAAGGCAGGGGCGCCAACGTGCTGCTCAGAGCCCTGGAGCCCCTGGAG
GGCCTGGAGACCATGAGACAGCTGAGAGCCACCCTGAGAGCTGCCACCGCCGCTAGAGTGCTCGCCGACAGAGAGCTGTGCAGCGGCC
CTAGCAAGCTGTGCCAAGCCCTGGCCATCAACAAGAGCTTCGATCAGAGAGACCTGGCCCAAGACGAGGCCGTGTGGCTGGAGAGAGG
CCCTCTGGAGCCTAGCGAACCCGCTGTGGTGGCCGCTGCCCGGGTGGGGGTCGGGCACGCCGGCGAGTGGGCTAGAAAGCCCCTGAG
ATTCTACGTGAGAGGCAGCCCCTGGGTGAGCGTGGTGGACAGAGTCGCCGAGAGGGACACCCAAGCCTCCGGCGGCAGCAAGAGAAC
AGCCGACGGCTCCGAGTTTGAACCCAAGAAAAAGAGAAAAGTGTAA

ACG-BE5

ATGAAACGGACAGCCGACGGAAGCGAGTTCGAGTCACCAAAGAAGAAGCGGAAAGTCTCTGAGGTGGAGTTTTCCCACGAGTACTGGA
TGAGACATGCCCTGACCCTGGCCAAGAGGGCACGGGATGAGAGGAGCGTGCCTGTGGGAGCCGTGCTGGTGCTGAACAATAGAGTGAT
CGGCGAGGGCTGGAACAGAGCCAAGGGCCTGCACGACCCAACAGCCCATGCCGAAATTATGGCCCTGAGACAGGGCGGCCTGGTCATG
CAGAACTACAGACTGTACGACGCCACCCTGTACACCACATTCGAGCCTTGCGTGATGTGCGCCGGCGCCATGATCCACTCTAGGATCGGCC

GCGTGGTGTTTGGCGTGAGGAACGCCAAAACCGGCGCCGCAGGCTCCCTGATGGACGTGCTGCACCACCCCGGCATGAATCACCGCGTC
GAAATTACCGAGGGAATCCTGGCAGATGAATGTGCCGCCCTGCTGTGCAGGTTCTTCCGGATGCCTAGAAGGGTGTTCAATGCTCAGAAG
AAGGCCCAGAGCTCCACCGACTCCGGAGGATCTAGCGGAGGCTCCTCTGGCTCTGAGACACCTGGCACAAGCGAGAGCGCAACACCTG
AAAGCAGCGGGGGCAGCAGCGGGGGGTCA___Cas9n___AGCGGCGGCTCCAAAAGAACCGCCGACGGCAGCGAATTCGAGCCCAA
GAAGAAACGGAAGGTGCTGGGCGGCGACAGCGGGGGGTCCGGGGGCTCCGGCGGGAGCGTGACCCCCGCCCTGCAGATGAAGAAGC
CCAAGCAGTTCTGCAGAAGAATGGGGCAGAAGAAGCAGAGACCTGCTCGGGCTGGGCAGCCTCACAGCAGCAGCGACGCCGCCCAAG
CTCCTGCCGAGCAGCCTCATAGCAGCAGCGACGCTGCTCAAGCCCCTTGCCCTAGAGAGCGGTGCCTGGGGCCTCCCACCACCCCCGGC
CCCTACAGAAGCATCTACTTCAGCAGCCCCAAGGGCCACCTGACAAGACTGGGCCTGGAGTTCTTCGATCAGCCCGCCGTGCCCCTGGCT
AGAGCCTTCCTGGGCCAAGTGCTGGTGAGAAGACTGCCCAACGGCACCGAGCTGAGAGGCAGAATCGTGGAGACCGAGGCCTACCTGG
GCCCCGAGGACGAAGCTGCCCACAGCAGAGGCGGCAGACAGACCCCTAGAAACAGAGGCATGTTCATGAAGCCCGGCACCCTGTACGT
GTACATCATCTACAGAATGTACTTCTGCATGGGCATCAGCAGCCAAGGCAGGGGCGCCAACGTGCTGCTCAGAGCCCTGGAGCCCCTGGA
GGGCCTGGAGACCATGAGACAGCTGAGAGCCACCCTGAGAGCTGCCACCGCCGCTAGAGTGCTCGCCGACAGAGAGCTGTGCAGCGGC
CCTAGCAAGCTGTGCCAAGCCCTGGCCATCAACAAGAGCTTCGATCAGAGAGACCTGGCCCAAGACGAGGCCGTGTGGCTGGAGAGAG
GCCCTCTGGAGCCTAGCGAACCCGCTGTGGTGGCCGCTGCCCGGGTGGGGGTCGGGCACGCCGGCGAGTGGGCTAGAAAGCCCCTGA
GATTCTACGTGAGAGGCAGCCCCTGGGTGAGCGTGGTGGACAGAGTCGCCGAGAGGGACACCCAAGCCTCCGGCGGCAGCAAGAGAA
CAGCCGACGGCTCCGAGTTTGAACCCAAGAAAAAGAGAAAAGTGTAA

ACG-BE6

ATGAAACGGACAGCCGACGGAAGCGAGTTCGAGTCACCAAAGAAGAAGCGGAAAGTCTCTGAGGTGGAGTTTTCCCACGAGTACTGGA
TGAGACATGCCCTGACCCTGGCCAAGAGGGCACGGGATGAGAGGAGCGTGCCTGTGGGAGCCGTGCTGGTGCTGAACAATAGAGTGAT
CGGCGAGGGCTGGAACAGAGCCATCGGCCTGCACGACCCAACAGCCCATGCCGAAATTATGGCCCTGAGACAGGGCGGCCTGGTCATG
CAGAACTACAGACTGTACGACGCCACCCTGTACACCACATTCGAGCCTTGCGTGATGTGCGCCGGCGCCATGATCCACTCTAGGATCGGCC
GCGTGGTGTTTGGCGTGAGGAACGCCAAAACCGGCGCCGCAGGCTCCCTGATGGACGTGCTGCACCACCCCGGCATGAATCACCGCGTC
GAAATTACCGAGGGAATCCTGGCAGATGAATGTGCCGCCCTGCTGTGCAGGTTCTTCCGGATGCCTAGAAGGGTGTTCAATGCTCAGAAG
AAGGCCCAGAGCTCCACCGACTCCGGAGGATCTAGCGGAGGCTCCTCTGGCTCTGAGACACCTGGCACAAGCGAGAGCGCAACACCTG
AAAGCAGCGGGGGCAGCAGCGGGGGGTCA___Cas9n___AGCGGCGGCTCCAAAAGAACCGCCGACGGCAGCGAATTCGAGCCCAA
GAAGAAACGGAAGGTGCTGGGCGGCGACAGCGGGGGGTCCGGGGGCTCCGGCGGGAGCGTGACCCCCGCCCTGCAGATGAAGAAGC
CCAAGCAGTTCTGCAGAAGAATGGGGCAGAAGAAGCAGAGACCTGCTCGGGCTGGGCAGCCTCACAGCAGCAGCGACGCCGCCCAAG
CTCCTGCCGAGCAGCCTCATAGCAGCAGCGACGCTGCTCAAGCCCCTTGCCCTAGAGAGCGGTGCCTGGGGCCTCCCACCACCCCCGGC
CCCTACAGAAGCATCTACTTCAGCAGCCCCAAGGGCCACCTGACAAGACTGGGCCTGGAGTTCTTCGATCAGCCCGCCGTGCCCCTGGCT
AGAGCCTTCCTGGGCCAAGTGCTGGTGAGAAGACTGCCCAACGGCACCGAGCTGAGAGGCAGAATCGTGGAGACCGAGGCCTACCTGG
GCCCCGAGGACGAAGCTGCCCACAGCAGAGGCGGCAGACAGACCCCTAGAAACAGAGGCATGTTCATGAAGCCCGGCACCCTGTACGT
GTACATCATCTACAGAATGTACTTCTGCATGGGCATCAGCAGCCAAGGCAGGGGCGCCAACGTGCTGCTCAGAGCCCTGGAGCCCCTGGA
GGGCCTGGAGACCATGAGACAGCTGAGAGCCACCCTGAGAGCTGCCACCGCCGCTAGAGTGCTCGCCGACAGAGAGCTGTGCAGCGGC
CCTAGCAAGCTGTGCCAAGCCCTGGCCATCAACAAGAGCTTCGATCAGAGAGACCTGGCCCAAGACGAGGCCGTGTGGCTGGAGAGAG
GCCCTCTGGAGCCTAGCGAACCCGCTGTGGTGGCCGCTGCCCGGGTGGGGGTCGGGCACGCCGGCGAGTGGGCTAGAAAGCCCCTGA
GATTCTACGTGAGAGGCAGCCCCTGGGTGAGCGTGGTGGACAGAGTCGCCGAGAGGGACACCCAAGCCTCCGGCGGCAGCAAGAGAA
CAGCCGACGGCTCCGAGTTTGAACCCAAGAAAAAGAGAAAAGTGTAA

ACG-BE7

ATGAAACGGACAGCCGACGGAAGCGAGTTCGAGTCACCAAAGAAGAAGCGGAAAGTCTCTGAGGTGGAGTTCAGCCACGAGTACTGG
ATGAGACACGCCCTGACCCTGGCTAAGAGGGCACGGGATGAGGGCGAGGCCCCTGTGGGAGCCGTGCTGGTGCTGAACAATAGAGTGA
TCGGCGAGGGCTGGAACAGAAGAATCGGCCTGCACGACCCAACAGCCCATGCCGAAATTATGGCCCTGAGACAGGGCGGCCTGGTCAT
GCAGAACAGCAGACTGATTGACGCCACCCTGTACGTGACATTCGAGCCTTGCGTGATGTGCGCCGGCGCCATGATCAACTCTAGGATCGG
CCGCGTGGTGTTTGGCGTGAGGAACTCAAAAAGAGGCGCCGCAGGCTCCCTGATGAACGTGCTGAACTACCCCGGCATGAATCACCGCG
TCGAAATTACCGAGGGAATCCTGGCAGATGAATGTGCCGCCCTGCTGTGCGATTTCTATCGGATGCCTAGACAGGTGTTCAATGCTCAGAA
GAAGGCCCAGAGCTCCATCAACTCCGGAGGATCTAGCGGAGGCTCCTCTGGCTCTGAGACACCTGGCACAAGCGAGAGCGCAACACCT
GAAAGCAGCGGGGGCAGCAGCGGGGGGTCA___Cas9n___AGCGGCGGCTCCAAAAGAACCGCCGACGGCAGCGAATTCGAGCCCA

AGAAGAAACGGAAGGTGCTGGGCGGCGACAGCGGGGGTCCGGGGCTCCGGCGGGAGCGTGACCCCGCCCTGCAGATGAAGAAG
CCCAAGCAGTTCTGCAGAAGAATGGGGCAGAAGAAGCAGAGACCTGCTCGGGCTGGGCAGCCTCACAGCAGCAGCGACGCCGCCCAA
GCTCCTGCCGAGCAGCCTCATAGCAGCAGCGACGCTGCTCAAGCCCCTTGCCCTAGAGAGCGGTGCCTGGGGCCTCCCACCACCCCCGG
CCCCTACAGAAGCATCTACTTCAGCAGCCCCAAGGGCCACCTGACAAGACTGGGCCTGGAGTTCTTCGATCAGCCCGCCGTGCCCCTGGC
TAGAGCCTTCCTGGGCCAAGTGCTGGTGAGAAGACTGCCCAACGGCACCGAGCTGAGAGGCAGAATCGTGGAGACCGAGGCCTACCTG
GGCCCCGAGGACGAAGCTGCCCACAGCAGAGGCGGCAGACAGACCCCTAGAAACAGAGGCATGTTCATGAAGCCCGGCACCCTGTACG
TGTACATCATCTACAGAATGTACTTCTGCATGGGCATCAGCAGCCAAGGCAGGGGCGCCAACGTGCTGCTCAGAGCCCTGGAGCCCCTGG
AGGGCCTGGAGACCATGAGACAGCTGAGAGCCACCCTGAGAGCTGCCACCGCCGCTAGAGTGCTCGCCGACAGAGAGCTGTGCAGCGG
CCCTAGCAAGCTGTGCCAAGCCCTGGCCATCAACAAGAGCTTCGATCAGAGAGACCTGGCCCAAGACGAGGCCGTGTGGCTGGAGAGA
GGCCCTCTGGAGCCTAGCGAACCCGCTGTGGTGGCCGCTGCCCGGGTGGGGGTCGGGCACGCCGGCGAGTGGGCTAGAAAGCCCCTG
AGATTCTACGTGAGAGGCAGCCCCTGGGTGAGCGTGGTGGACAGAGTCGCCGAGAGGGACACCCAAGCCTCCGGCGGCAGCAAGAGA
ACAGCCGACGGCTCCGAGTTTGAACCCAAGAAAAAGAGAAAAGTGTAA

ACG-BE7-MT(N)

ATGAAACGGACAGCCGACGGAAGCGAGTTCGAGTCACCAAAGAAGAAGCGGAAAGTCGTGACCCCGCCCTGCAGATGAAGAAGCCC
AAGCAGTTCTGCAGAAGAATGGGGCAGAAGAAGCAGAGACCTGCTCGGGCTGGGCAGCCTCACAGCAGCAGCGACGCCGCCCAAGCT
CCTGCCGAGCAGCCTCATAGCAGCAGCGACGCTGCTCAAGCCCCTTGCCCTAGAGAGCGGTGCCTGGGGCCTCCCACCACCCCCGGCCC
CTACAGAAGCATCTACTTCAGCAGCCCCAAGGGCCACCTGACAAGACTGGGCCTGGAGTTCTTCGATCAGCCCGCCGTGCCCCTGGCTAG
AGCCTTCCTGGGCCAAGTGCTGGTGAGAAGACTGCCCAACGGCACCGAGCTGAGAGGCAGAATCGTGGAGACCGAGGCCTACCTGGGC
CCCGAGGACGAAGCTGCCCACAGCAGAGGCGGCAGACAGACCCCTAGAAACAGAGGCATGTTCATGAAGCCCGGCACCCTGTACGTGT
ACATCATCTACAGAATGTACTTCTGCATGGGCATCAGCAGCCAAGGCAGGGGCGCCAACGTGCTGCTCAGAGCCCTGGAGCCCCTGGAG
GGCCTGGAGACCATGAGACAGCTGAGAGCCACCCTGAGAGCTGCCACCGCCGCTAGAGTGCTCGCCGACAGAGAGCTGTGCAGCGGCC
CTAGCAAGCTGTGCCAAGCCCTGGCCATCAACAAGAGCTTCGATCAGAGAGACCTGGCCCAAGACGAGGCCGTGTGGCTGGAGAGAGG
CCCTCTGGAGCCTAGCGAACCCGCTGTGGTGGCCGCTGCCCGGGTGGGGGTCGGGCACGCCGGCGAGTGGGCTAGAAAGCCCCTGAG
ATTCTACGTGAGAGGCAGCCCCTGGGTGAGCGTGGTGGACAGAGTCGCCGAGAGGGACACCCAAGCCGAGGCCGCCGCCAAGGAAGC
TGCCGCCAAGGAGGCCGCCGCCAAGTCTGAGGTGGAGTTCAGCCACGAGTACTGGATGAGACACGCCCTGACCCTGGCTAAGAGGGCA
CGGGATGAGGGCGAGGCCCCTGTGGGAGCCGTGCTGGTGCTGAACAATAGAGTGATCGGCGAGGGCTGGAACAGAAGAATCGGCCTG
CACGACCCAACAGCCCATGCCGAAATTATGGCCCTGAGACAGGGCGGCCTGGTCATGCAGAACAGCAGACTGATTGACGCCACCCTGTAC
GTGACATTCGAGCCTTGCGTGATGTGCGCCGGCGCCATGATCAACTCTAGGATCGGCCGCGTGGTGTTTGGCGTGAGGAACTCAAAAAG
AGGCGCCGCAGGCTCCCTGATGAACGTGCTGAACTACCCCGGCATGAATCACCGCGTCGAAATTACCGAGGGAATCCTGGCAGATGAATG
TGCCGCCCTGCTGTGCGATTTCTATCGGATGCCTAGACAGGTGTTCAATGCTCAGAAGAAGGCCCAGAGCTCCATCAACTCCGGAGGATCT
AGCGGAGGCTCCTCTGGCTCTGAGACACCTGGCACAAGCGAGAGCGCAACACCTGAAAGCAGCGGGGGCAGCAGCGGGGGGTCA___
Cas9n___ TCCGGCGGCAGCAAGAGAACAGCCGACGGCTCCGAGTTTGAACCCAAGAAAAAGAGAAAAGTGTAA

ACG-BE7-TM(N)

ATGAAACGGACAGCCGACGGAAGCGAGTTCGAGTCACCAAAGAAGAAGCGGAAAGTCTCTGAGGTGGAGTTCAGCCACGAGTACTGG
ATGAGACACGCCCTGACCCTGGCTAAGAGGGCACGGGATGAGGGCGAGGCCCCTGTGGGAGCCGTGCTGGTGCTGAACAATAGAGTGA
TCGGCGAGGGCTGGAACAGAAGAATCGGCCTGCACGACCCAACAGCCCATGCCGAAATTATGGCCCTGAGACAGGGCGGCCTGGTCAT
GCAGAACAGCAGACTGATTGACGCCACCCTGTACGTGACATTCGAGCCTTGCGTGATGTGCGCCGGCGCCATGATCAACTCTAGGATCGG
CCGCGTGGTGTTTGGCGTGAGGAACTCAAAAAGAGGCGCCGCAGGCTCCCTGATGAACGTGCTGAACTACCCCGGCATGAATCACCGCG
TCGAAATTACCGAGGGAATCCTGGCAGATGAATGTGCCGCCCTGCTGTGCGATTTCTATCGGATGCCTAGACAGGTGTTCAATGCTCAGAA
GAAGGCCCAGAGCTCCATCAACGAGGCCGCCGCCAAGGAAGCTGCCGCCAAGGAGGCCGCCGCCAAGGTGACCCCGCCCTGCAGATG
AAGAAGCCCAAGCAGTTCTGCAGAAGAATGGGGCAGAAGAAGCAGAGACCTGCTCGGGCTGGGCAGCCTCACAGCAGCAGCGACGCC
GCCCAAGCTCCTGCCGAGCAGCCTCATAGCAGCAGCGACGCTGCTCAAGCCCCTTGCCCTAGAGAGCGGTGCCTGGGGCCTCCCACCAC
CCCCGGCCCCTACAGAAGCATCTACTTCAGCAGCCCCAAGGGCCACCTGACAAGACTGGGCCTGGAGTTCTTCGATCAGCCCGCCGTGCC
CCTGGCTAGAGCCTTCCTGGGCCAAGTGCTGGTGAGAAGACTGCCCAACGGCACCGAGCTGAGAGGCAGAATCGTGGAGACCGAGGCC
TACCTGGGCCCCGAGGACGAAGCTGCCCACAGCAGAGGCGGCAGACAGACCCCTAGAAACAGAGGCATGTTCATGAAGCCCGGCACCC
TGTACGTGTACATCATCTACAGAATGTACTTCTGCATGGGCATCAGCAGCCAAGGCAGGGGCGCCAACGTGCTGCTCAGAGCCCTGGAGC

CCCTGGAGGGCCTGGAGACCATGAGACAGCTGAGAGCCACCCTGAGAGCTGCCACCGCCGCTAGAGTGCTCGCCGACAGAGAGCTGTG
CAGCGGCCCTAGCAAGCTGTGCCAAGCCCTGGCCATCAACAAGAGCTTCGATCAGAGAGACCTGGCCCAAGACGAGGCCGTGTGGCTG
GAGAGAGGCCCTCTGGAGCCTAGCGAACCCGCTGTGGTGGCCGCTGCCCGGGTGGGGGTCGGGCACGCCGGCGAGTGGGCTAGAAAG
CCCCTGAGATTCTACGTGAGAGGCAGCCCCTGGGTGAGCGTGGTGGACAGAGTCGCCGAGAGGGACACCCAAGCCTCCGGAGGATCTA
GCGGAGGCTCCTCTGGCTCTGAGACACCTGGCACAAGCGAGAGCGCAACACCTGAAAGCAGCGGGGGCAGCAGCGGGGGGTCA___C
as9n___ TCCGGCGGCAGCAAGAGAACAGCCGACGGCTCCGAGTTTGAACCCAAGAAAAAGAGAAAAGTGTAA
