# Supplementary material for: Triple base editor catalyzes saturation mutation of adenine, cytidine, and guanine
Source: Nucleic Acids Res. 2026 Jan 14;54(2):gkaf1423. doi: 10.1093/nar/gkaf1423 (PMC12802905; doi:10.1093/nar/gkaf1423)

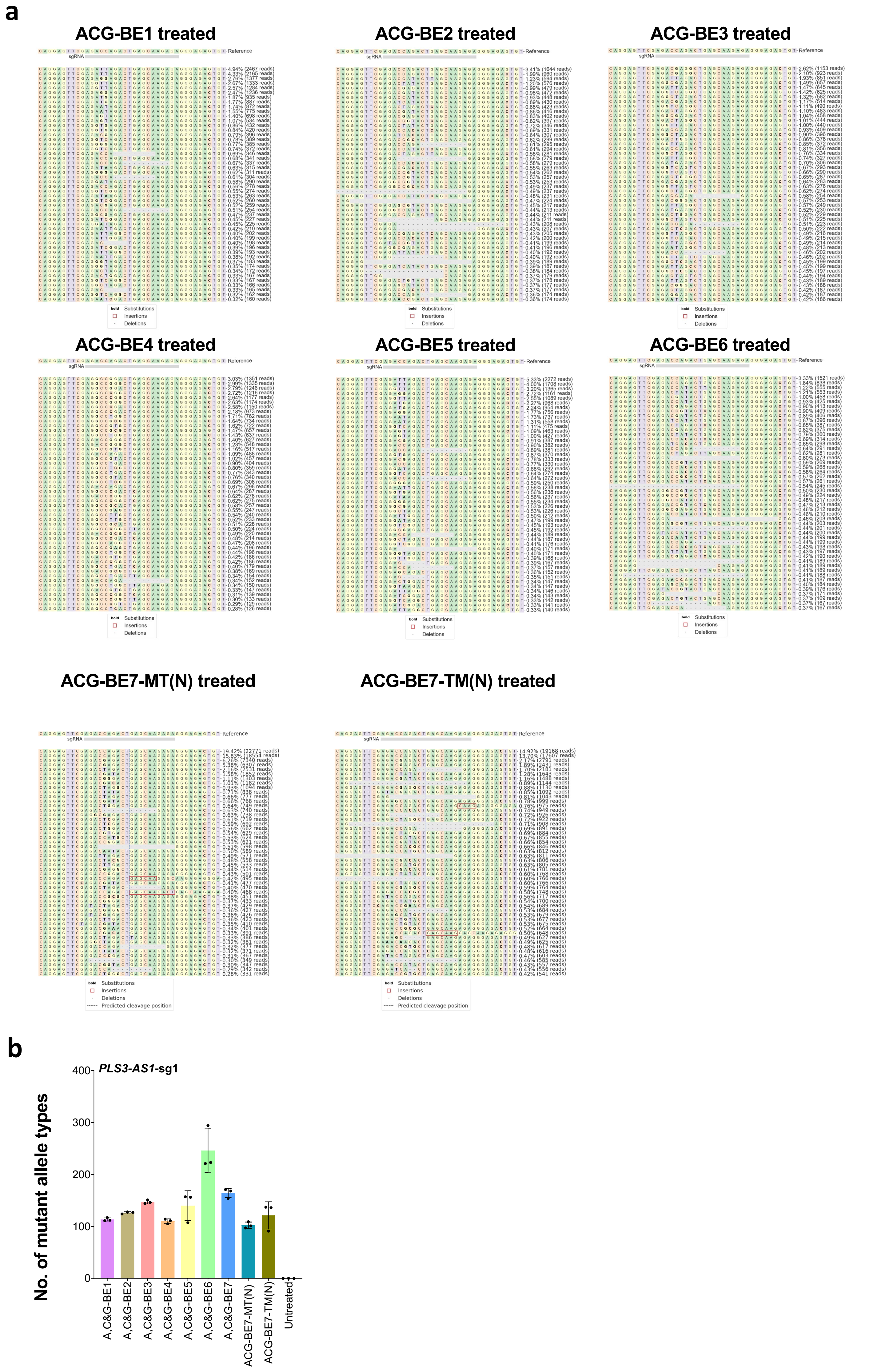

Supplementary Fig.1 Screen of triple base editors (ACG-BEs). **a**, Allele table for *PLS3-AS1*-sg1in HEK293T cells after ACG-BE1-6 transfection. The target site allele is boxed in a gray line. The percentile and sequencing reads of each allele at one representative of three independent experiments are listed on the right. **b**, Mutant allele types induced by ACG-BEs at *PLS3-AS1*-sg1.

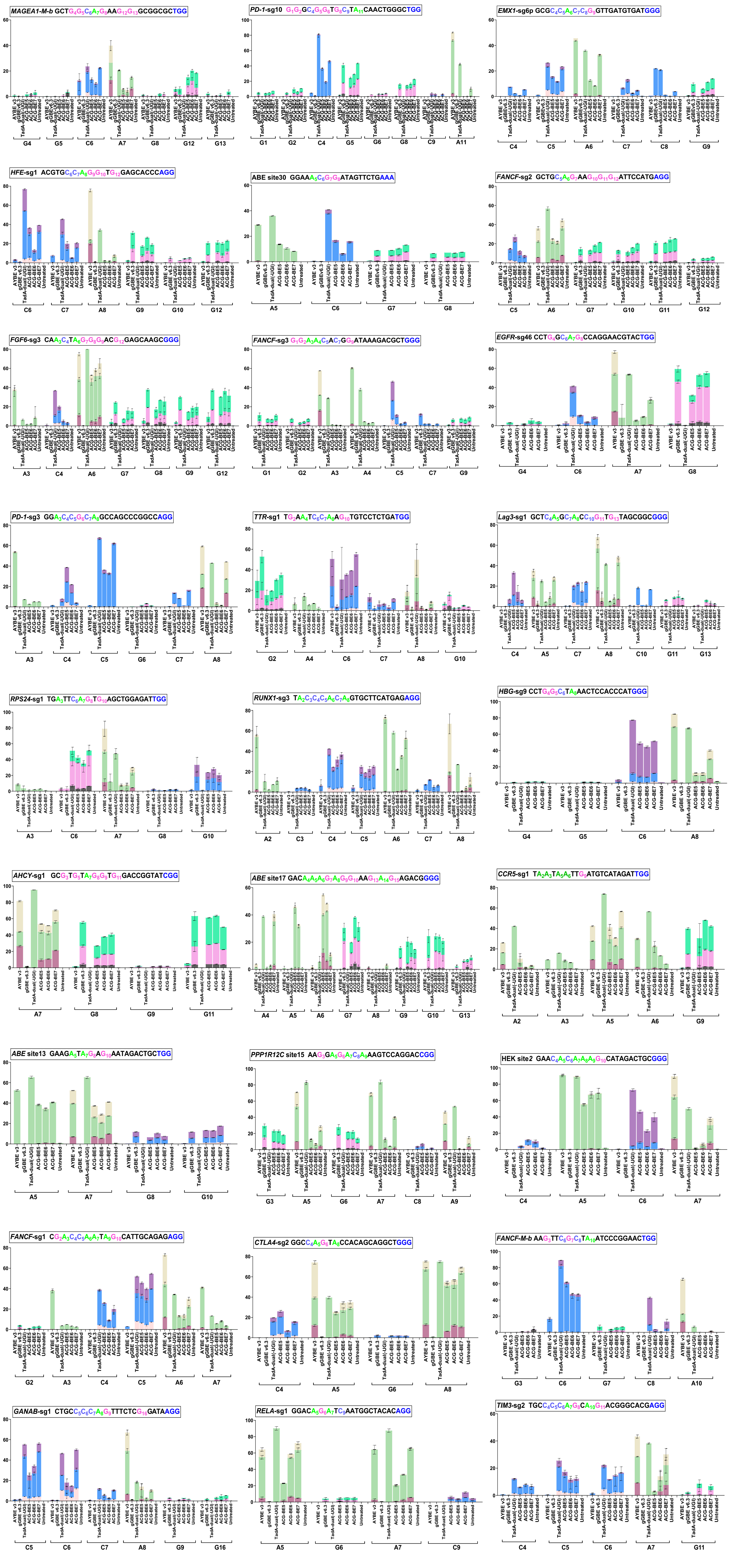

Supplementary Fig.2 Characterization of triple base editors (ACG-BEs). Base editing outcome of ACG-BEs at the 27 endogenous target in HEK293T cells. Data are means ± SD (n = 3 independent experiments).

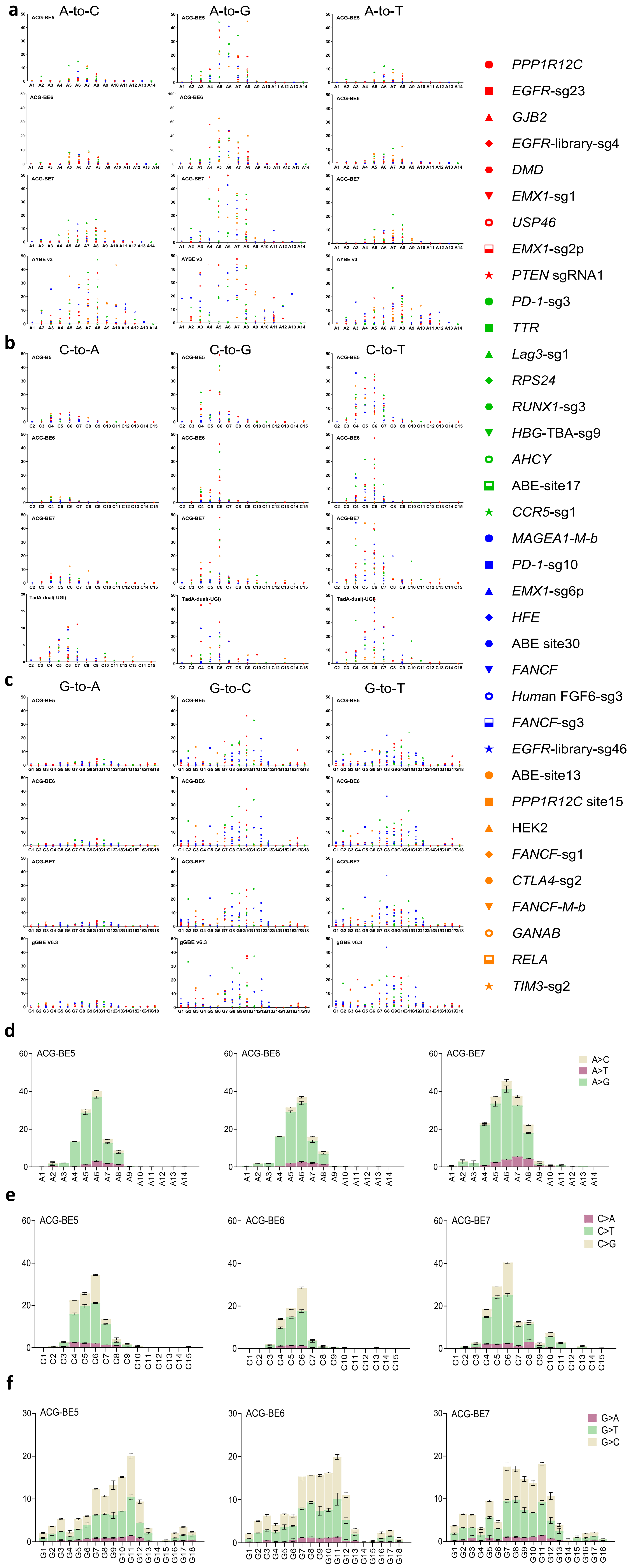

Supplementary Fig.3 The characteristics of ACG-BEs in HEK293T cells. **a,** The editing efficiency and window of A-to-C/G/T induced by ACG-BEs at the 36 endogenous targets in HEK293T cells. **b,** The editing efficiency and window of C-to-A/G/T induced by ACG-BEs at the 36 endogenous targets in HEK293T cells. **c,** The editing efficiency and window of G-to-A/C/T induced by ACG-BEs at the 36 endogenous targets in HEK293T cells. **d,** The average editing efficiency of A-to-C/G/T induced by ACG-BEs across protospacer calculated from 36 endogenous targets in HEK293T cells. Data are means ± SD (n = 3 independent experiments). **e,** The average editing efficiency of C-to-A/G/T induced by ACG-BEs across protospacer calculated from 36 endogenous targets in HEK293T cells. Data are means ± SD (n = 3 independent experiments).**f,** The average editing efficiency of G-to-A/C/T induced by ACG-BEs across protospacer calculated from 36 endogenous targets in HEK293T cells. Data are means ± SD (n = 3 independent experiments).For a-c, Each data point represents means at indicated target sites from 3 independent experiments.

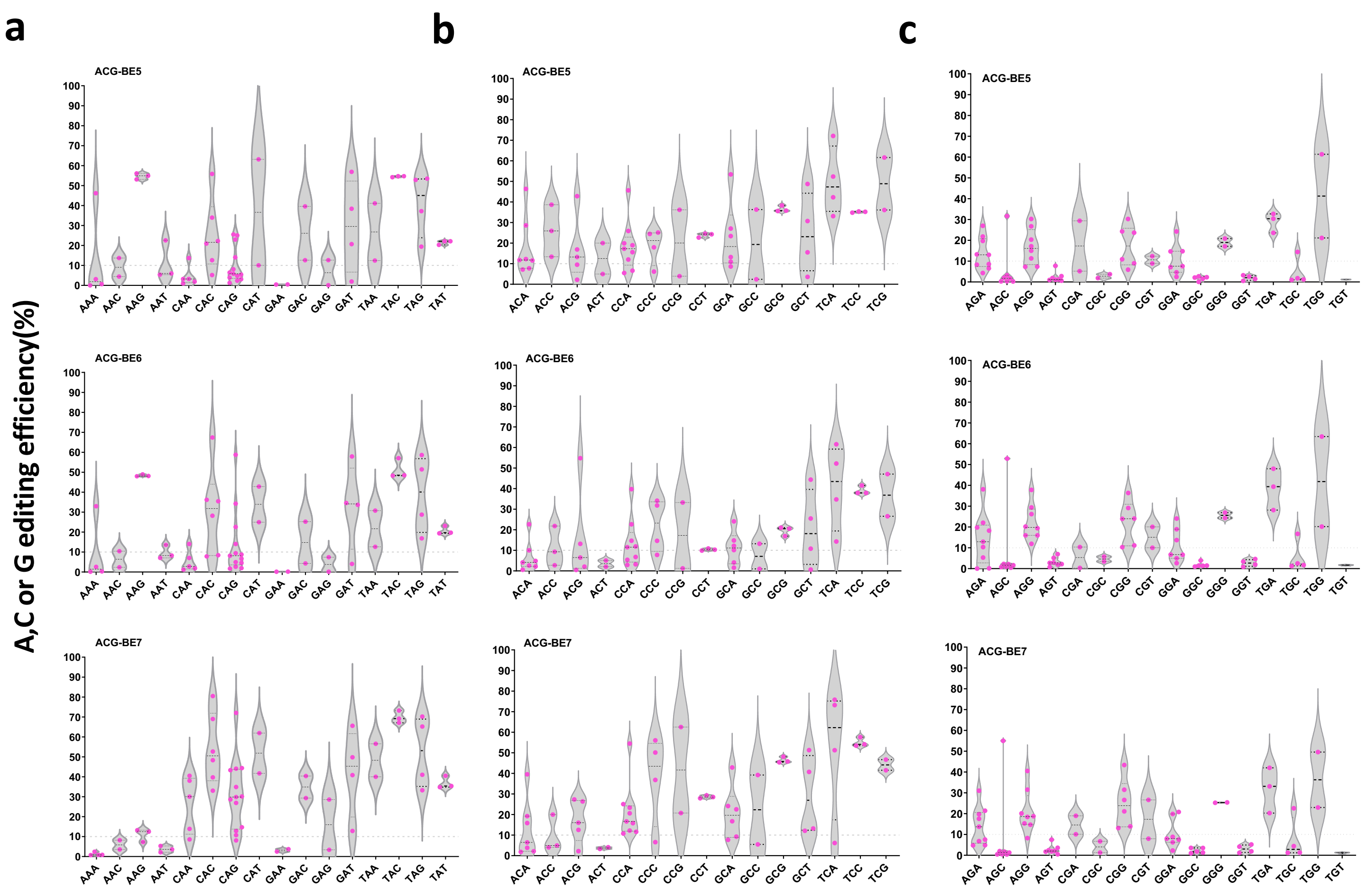

Supplementary Fig.4 The characteristics of ACG-BEs in HEK293T. **a,** The motif preference of A-to-C/G/T induced by ACG-BEs at the 36 endogenous targets in HEK293T cells. **b,** The motif preference of C-to-A/G/T induced by ACG-BEs at the 36 endogenous targets in HEK293T cells. **c,** The motif preference of G-to-A/C/T induced by ACG-BEs at the 36 endogenous targets in HEK293T cells. For a-c, Each data point represents means at indicated target sites from 3 independent experiments

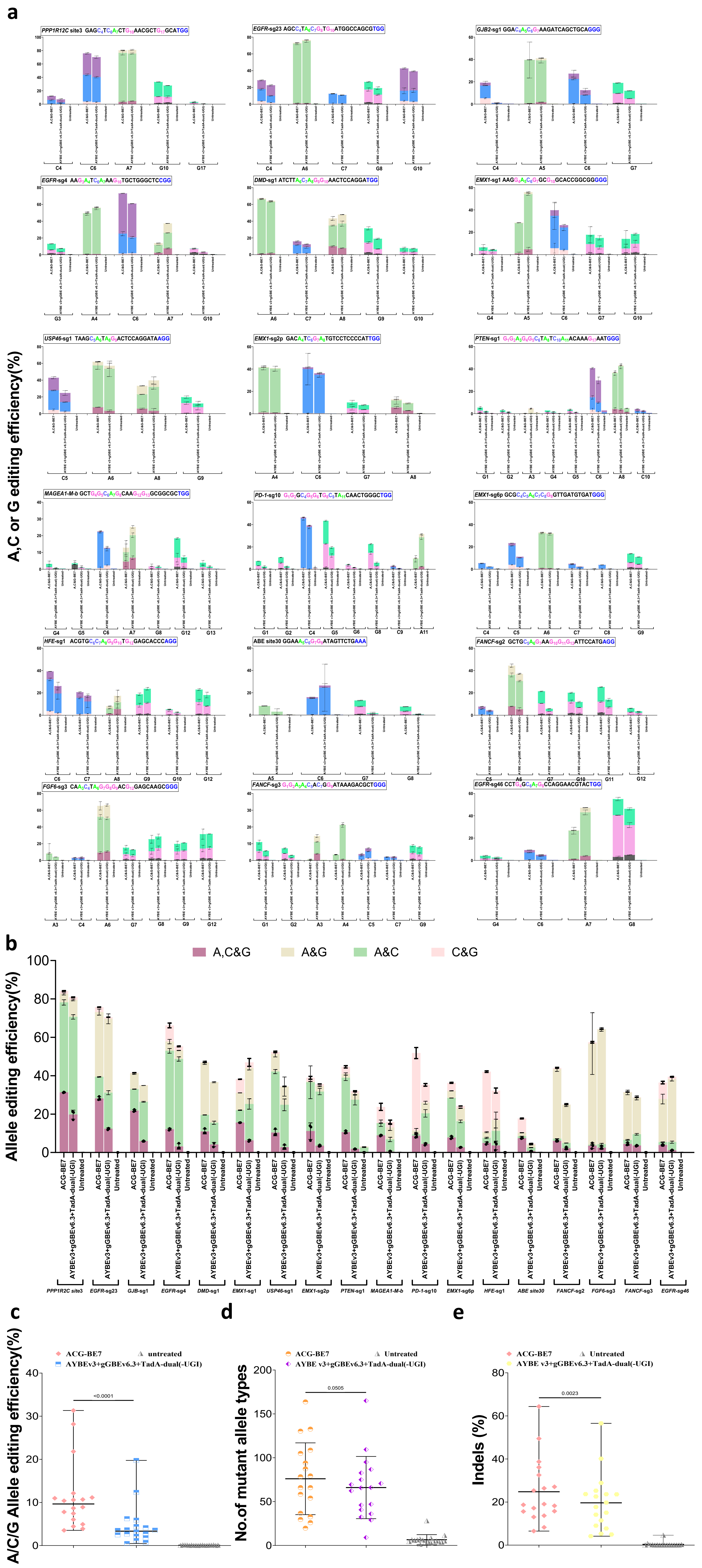

Supplementary Fig.5 Comparison of the editing efficiency induced by ACG-BE7 and the mixture of AYBE v3, TadA-dual(-UGI) and gGBE v6.3 in HEK293T cells. **a,** Base editing induced by ACG-BE7 and the mixture of AYBE v3, TadA-dual(-UGI) and gGBE v6.3 at the 18 endogenous targets in HEK293T cells. Data are means ± SD (n = 3 independent experiments). **b,** Allele base editing of A,C&G, A&G, A&C and C&G induced by ACG-BE7 and the mixture of AYBE v3, TadA-dual(-UGI) and gGBE v6.3 at the 18 endogenous targets in HEK293T cells. Data are means ± SD (n = 3 independent experiments). **c,** Summary of the A/C/G simultaneous editing induced by ACG-BEs at the 18 endogenous targets in HEK293T cells. **d**, Summary of the number of mutant allele types induced by ACG-BE7 and the mixture of AYBE v3, TadA-dual(-UGI) and gGBE v6.3 at the 18 endogenous targets in HEK293T cells. **e,** Summary of the indels induced by ACG-BE7 and the mixture of AYBE v3, TadA-dual(-UGI) and gGBE v6.3 at the 18 endogenous targets in HEK293T cells. For c-e, Each data point represents means at indicated target sites from 3 independent experiments. Significance was tested with paired two-sided Wilcoxon rank-sum test (c-e).

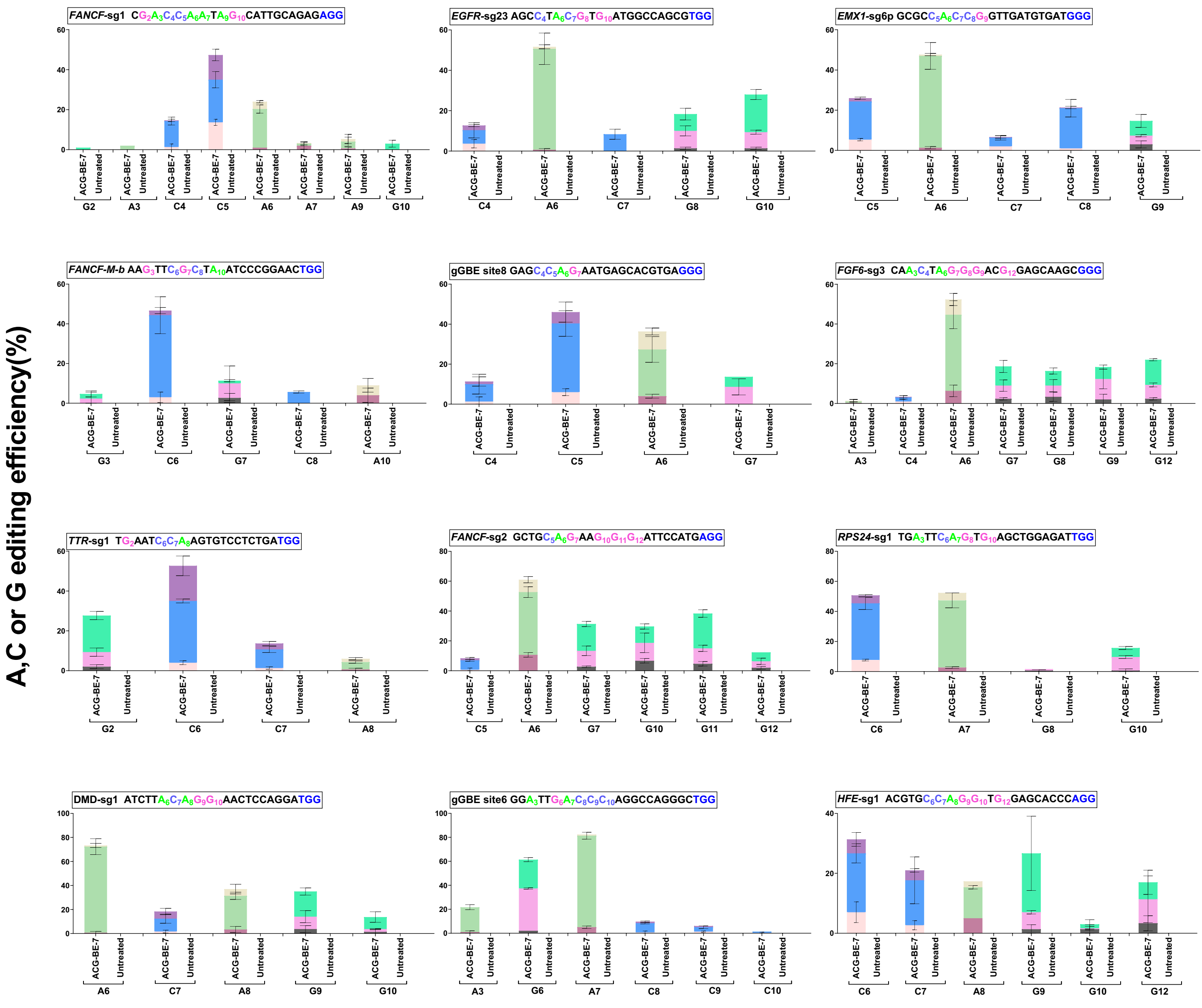

Supplementary Fig.6 Base editing outcome of ACG-BEs at the 12 endogenous target in HeLa cells. Data are means ± SD (n = 3 independent experiments).

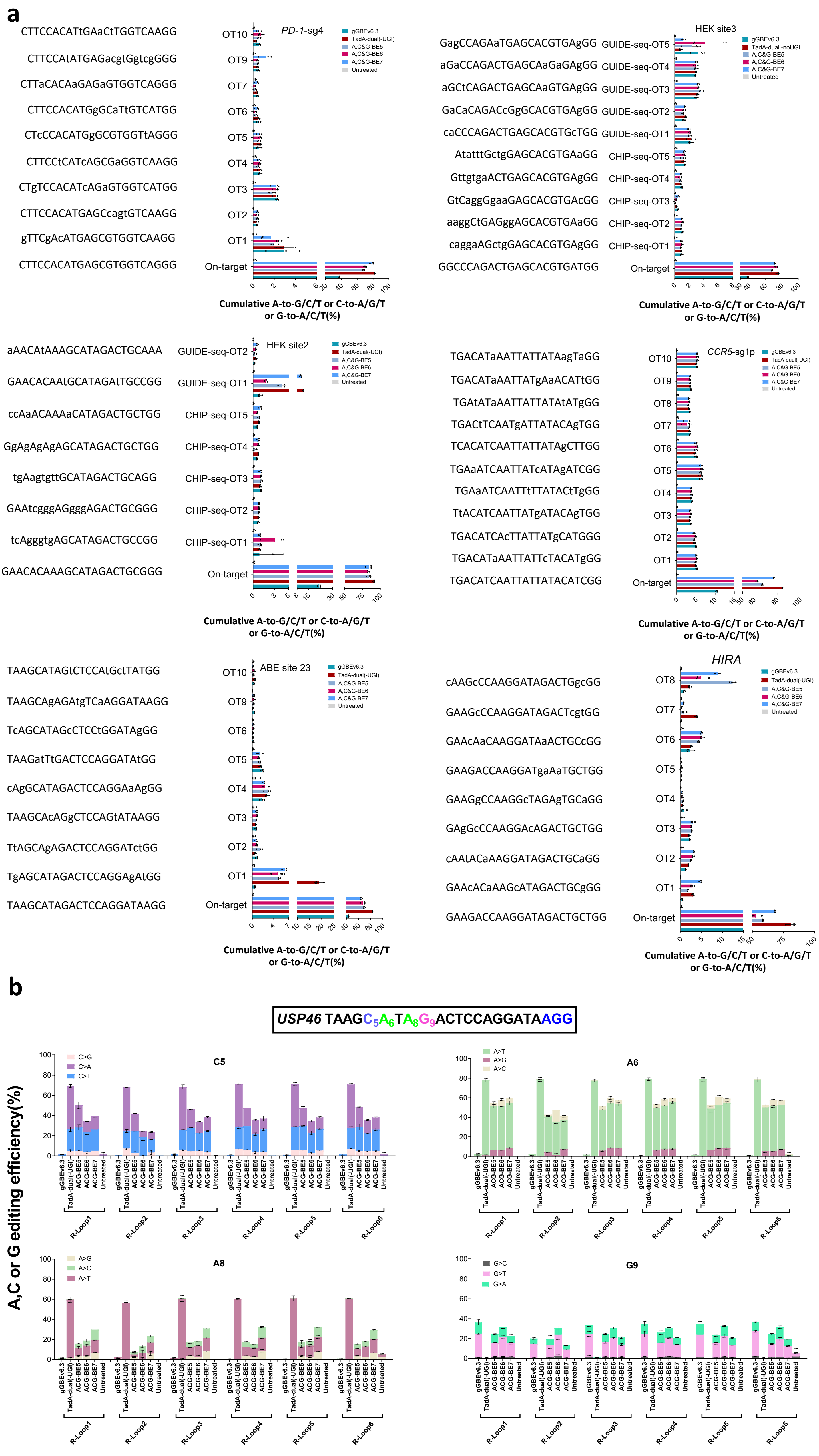

Supplementary Fig.7 Off-target evaluation of ACG-BEs. **a,** sgRNA-dependent DNA on- and off-target analysis of the indicated targets (*PD-1*-sg4, HEK site2,HEK site3, *CCR5*-sg1p, ABE site23 and *HIRA*) by ACG-BEs in HEK293T cells. Lowercase protospacer sequences represent mismatched bases compared to their corresponding on-target sequences. Data are means ± SD (n = 3 independent experiments). **b,** On-target base editing induced by ACG-BEs at the endogenous target (*USP46*) in HEK293T cells for orthogonal R-loop assay. Data are means ± SD (n = 3 independent experiments).

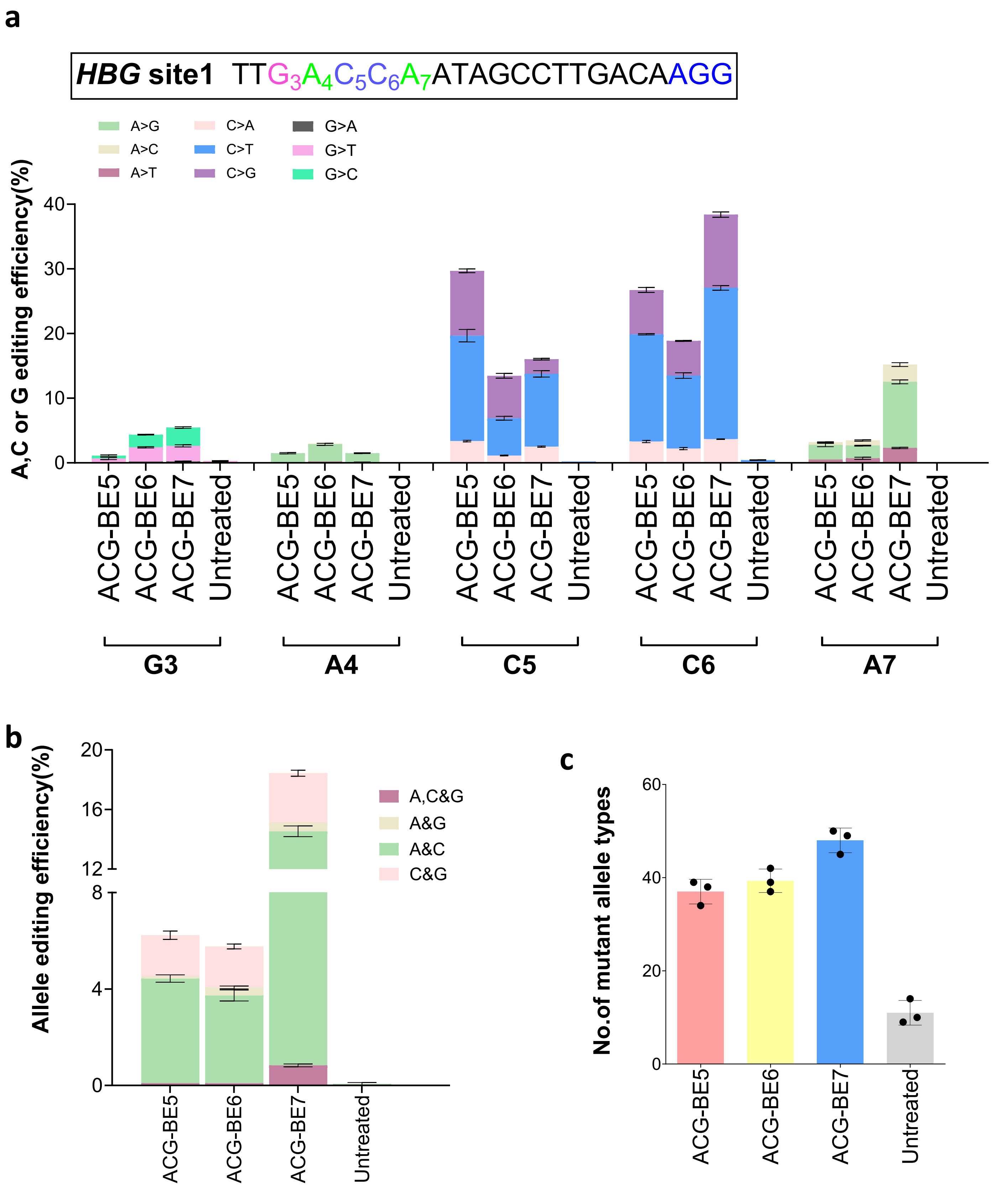

Supplementary Fig.8 Base editing induced by ACG-BEs at the endogenous target (*HBG* site1) in HEK293T cells. **a,** Base editing induced by ACG-BEs at *HBG* site1 in HEK293T cells. Data are means ± SD (n = 3 independent experiments). **b,** Allele base editing of A,C&G, A&G, A&C and C&G induced by ACG-BEs at *HBG* site1 in HEK293T cells. Data are means ± SD (n = 3 independent experiments). **c,** The number of mutant allele types induced by ACG-BEs at *HBG* site1 in HEK293T cells. Data are means ± SD (n = 3 independent experiments).

a

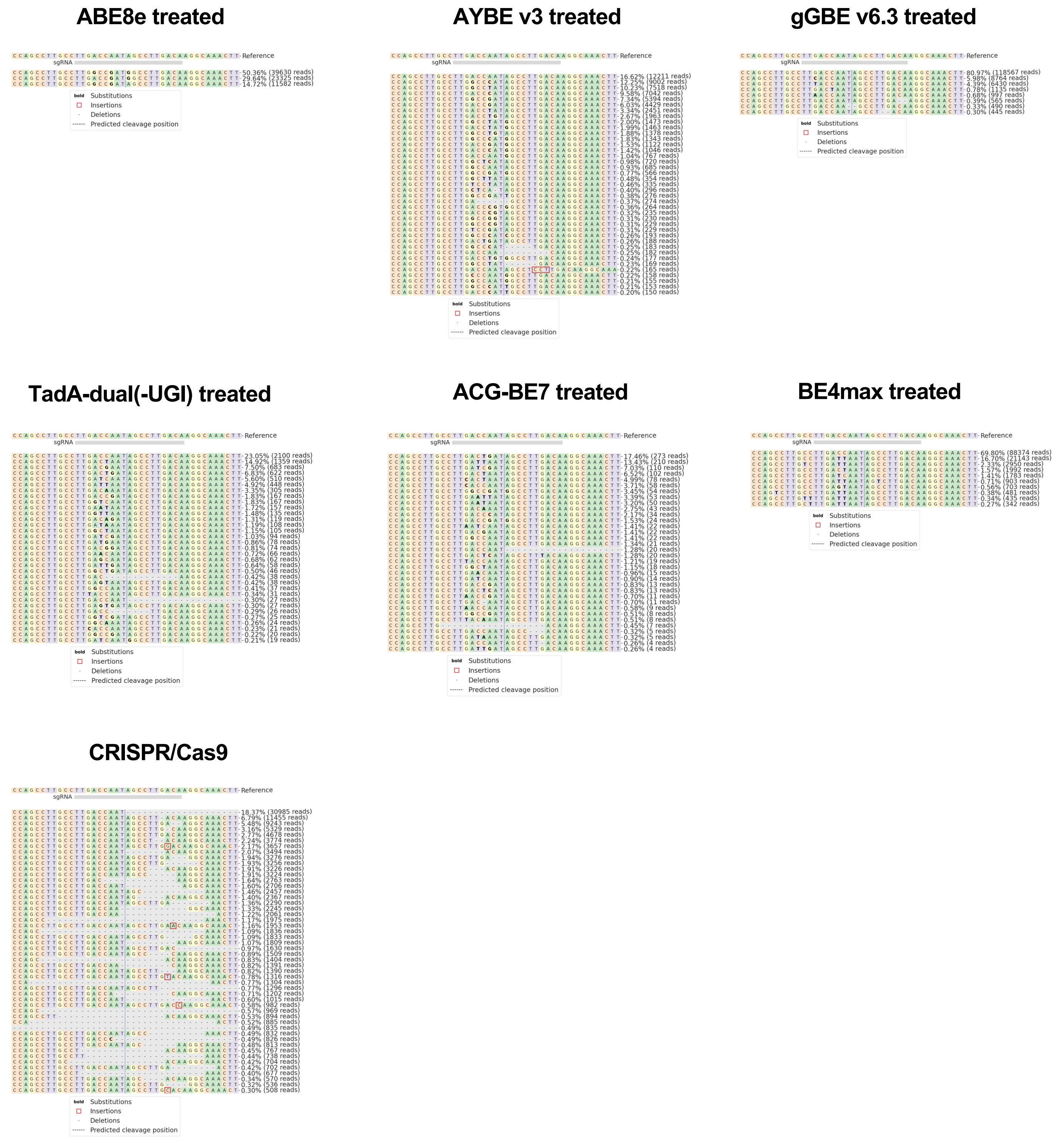

b

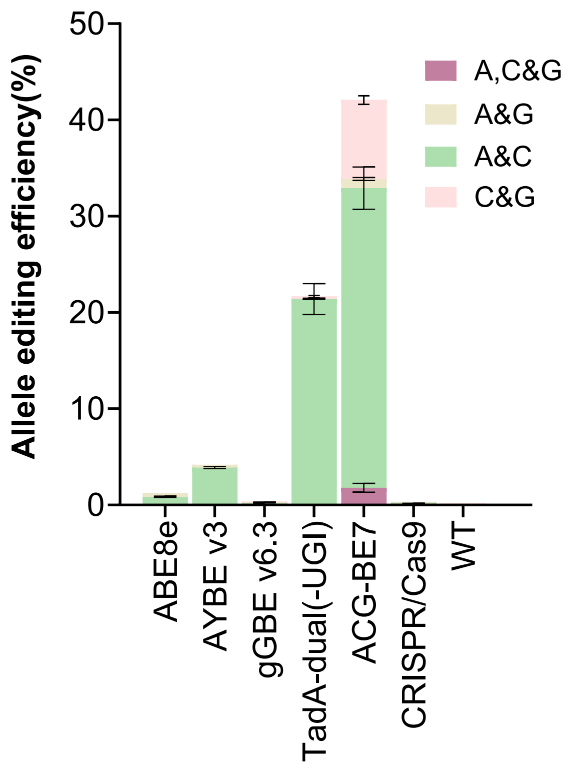

c

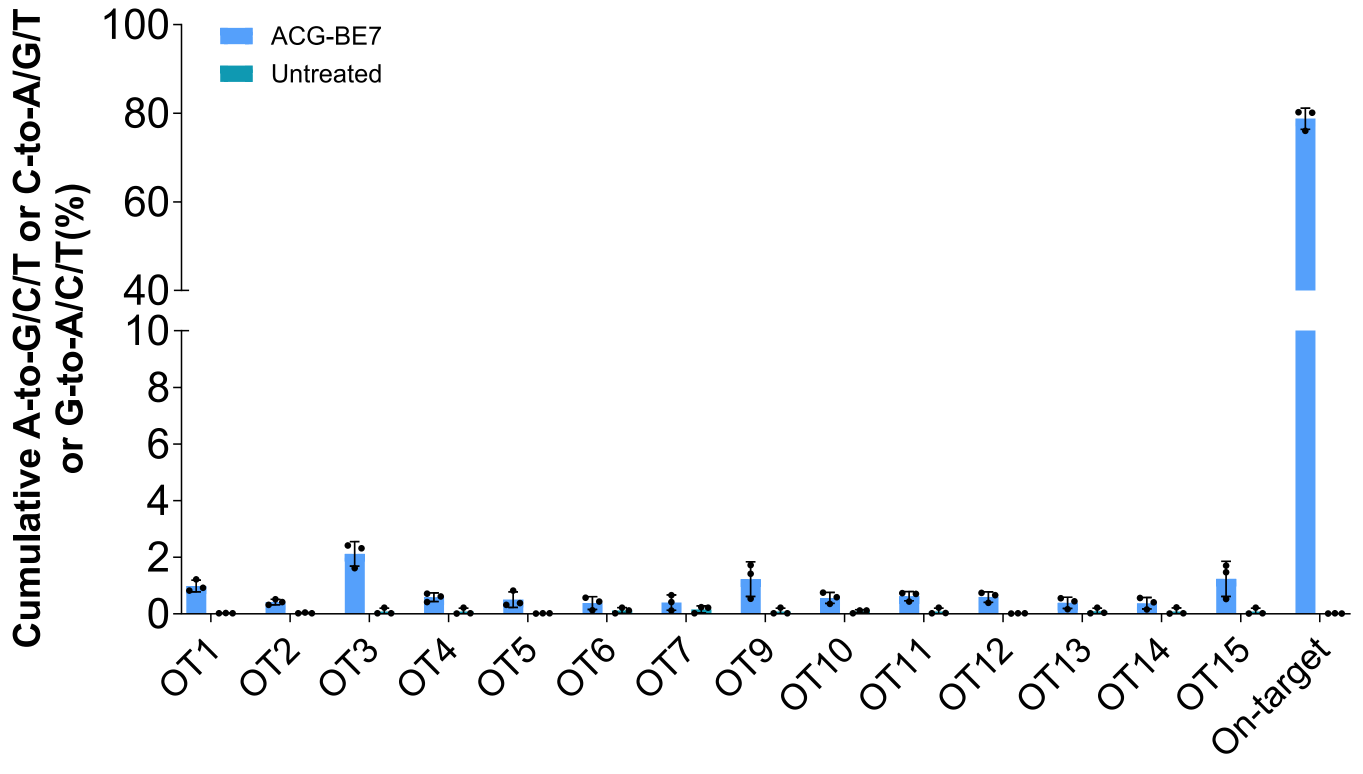

Supplementary Fig.9 Allele editing for *HBG* site1 in HUDEP-2($\Delta^{G}\gamma$) cells **a,** Allele table after base editors or CRISPR/Cas9 transfection. Target site allele is boxed in gray line. The percentile and sequencing reads of each allele at one representative of three independent experiments are listed on the right. **b,** Allele editing of A,C&G, A&G, A&C and C&G induced by ACG-BEs. Data are means ± SD (n = 3 independent experiments). **c**, sgRNA-dependent DNA on- and off-target analysis of the indicated targets (*HBG* site1) by ACG-BE7 in HUDEP-2($\Delta^{G}\gamma$) cells. Data are means ± SD (n = 3 independent experiments).

Codon and amino acids changes potentially generated by ACG-BEs

A, the number of codons changes after this codon can only be edited by ACG-BEs (at least two types of base substrates are edited, not including A-to-G and C-to-T simultaneous editing)

B, the number of the amino acid changes after editing by only ACG-BEs (at least two types of base substrates are edited, not including A-to-G and C-to-T simultaneous editing)

C, the number of codons changes after this codon can be edited by ACG-BEs

D, the number of the amino acid changes after editing by ACG-BEs

A Amino acid codon B		
A	Amino acid (codon)	B
C		D

	T	C	A	G	
T	0 0 Phe TTT 0 0	0 0 Ser TCT 3 3	0 0 Tyr TAT 3 3	0 0 Cys TGT 3 3	T
	0 0 Phe TTC 3 1	0 0 Ser TCC 15 5	8 4 Tyr TAC 15 5	9 4 Cys TGC 15 5	C
	0 0 Leu TTA 3 1	0 0 Ser TCA 15 5	0 0 * TAA 15 6	9 4 * TGA 15 6	A
	0 0 Leu TTG 3 1	9 5 Ser TCG 15 5	9 4 * TAG 15 6	0 0 Trp TGG 15 5	G
C	0 0 Leu CTT 3 3	0 0 Pro CCT 15 14	8 7 His CAT 15 14	9 9 Arg CGT 15 14	T
	0 0 Leu CTC 15 4	0 0 Pro CCC 63 19	42 12 His CAC 63 19	45 15 Arg CGC 63 19	C
	8 4 Leu CTA 15 4	42 19 Pro CCA 63 19	42 16 Gln CAA 63 19	53 18 Arg CGA 63 19	A
	9 3 Leu CTG 15 4	45 17 Pro CCG 63 19	53 17 Gln CAG 63 19	45 15 Arg CGG 63 19	G
A	0 0 Ile ATT 3 3	8 8 Thr ACT 15 14	0 0 Asn AAT 15 14	9 8 Ser AGT 15 14	T
	8 3 Ile ATC 15 4	42 15 Thr ACC 63 19	42 17 Asn AAC 63 19	53 19 Ser AGC 63 19	C
	0 0 Ile ATA 15 4	42 16 Thr ACA 63 19	0 0 Lys AAA 63 19	45 16 Arg AGA 63 19	A
	9 3 Met ATG 15 4	53 18 Thr ACG 63 19	45 16 Lys AAG 63 19	45 13 Arg AGG 63 19	G
G	0 0 Val GTT 3 3	9 9 Ala GCT 15 14	9 8 Asp GAT 15 14	0 0 Gly GGT 15 14	T
	9 4 Val GTC 15 4	45 15 Ala GCC 63 19	53 18 Asp GAC 63 19	45 19 Gly GGC 63 19	C
	9 4 Val GTA 15 4	53 19 Ala GCA 63 19	45 15 Glu GAA 63 19	45 19 Gly GGA 63 19	A
	0 0 Val GTG 15 4	45 18 Ala GCG 63 19	45 13 Glu GAG 63 19	0 0 Gly GGG 63 19	G

Supplementary Fig.10 Theoretical statistics of codon and amino acid changes induced by ACG-BEs. This analysis summarizes the predicted numbers of nucleotide substitutions and corresponding amino acid changes generated by ACG-BEs across all 64 codons.

Supplementary Note 1. FACS gating examples for GFP cell sorting conditions in transcriptome profiling.

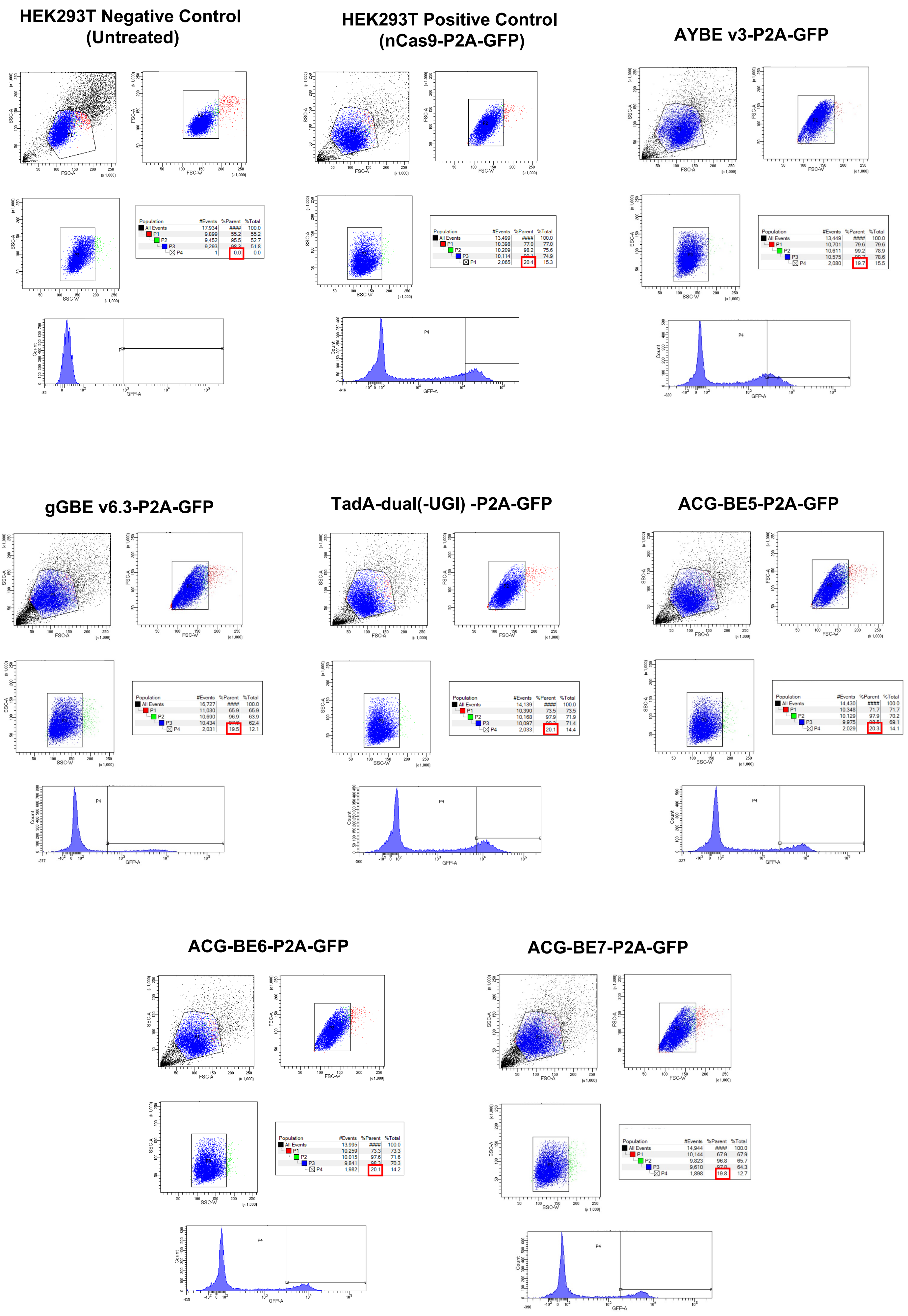

Supplementary Note 2. FACS gating examples for GFP cell sorting conditions in HUDEP-2 cells profiling.

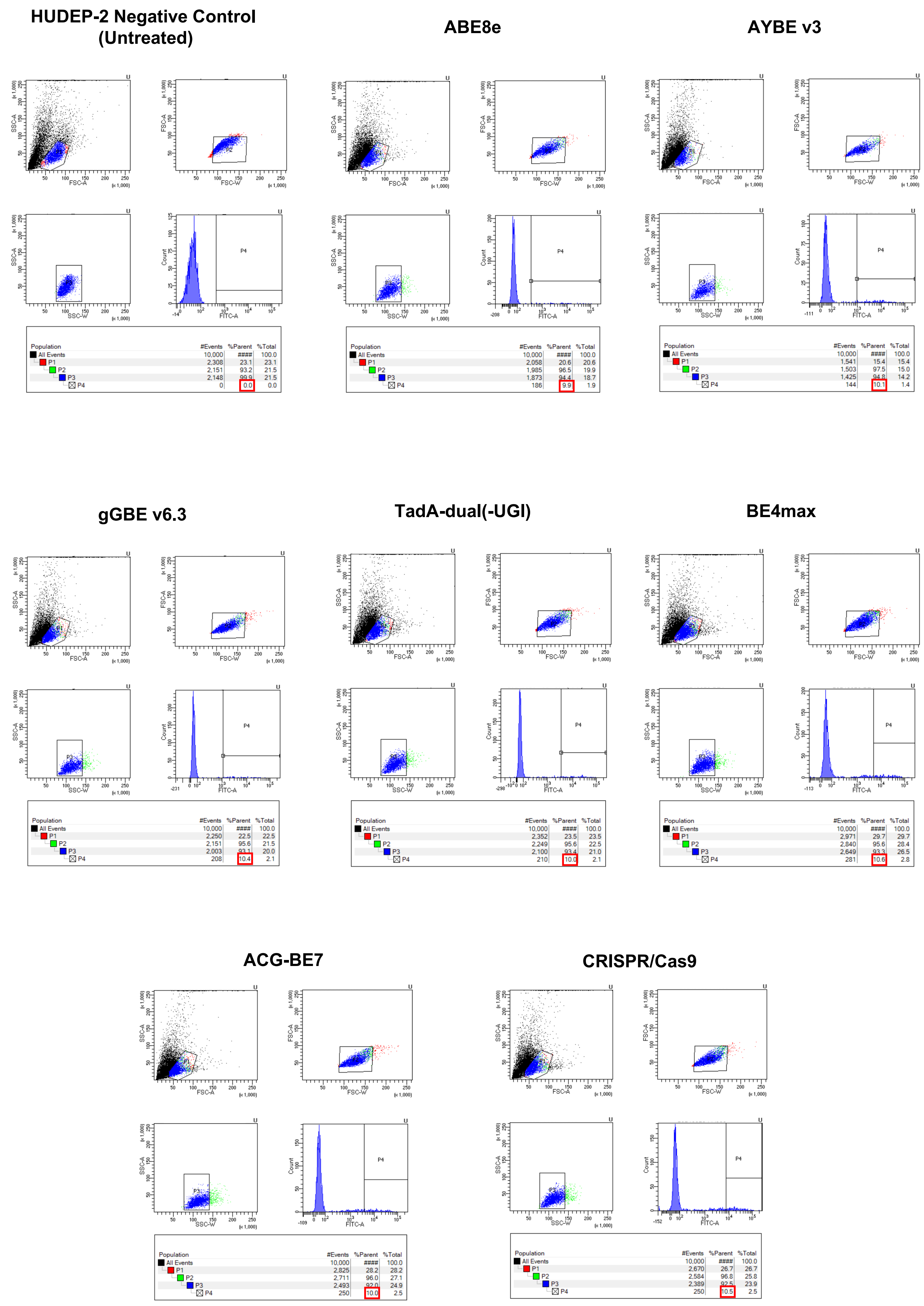

Supplement: gkaf1423_Supplemental_Files [file gkaf1423_supplemental_files.zip › Supplementary-Figure_251120-1505.pdf]
